# A novel genetic model provides a unique perspective on the relationship between postexercise glycogen concentration and increases in the abundance of key metabolic proteins after acute exercise

**DOI:** 10.1371/journal.pone.0295964

**Published:** 2024-01-30

**Authors:** Seong Eun Kwak, Amy Zheng, Edward B. Arias, Haiyan Wang, Xiufang Pan, Yongping Yue, Dongsheng Duan, Gregory D. Cartee

**Affiliations:** 1 Muscle Biology Laboratory, School of Kinesiology, University of Michigan, Ann Arbor, Michigan, United States of America; 2 Department of Molecular Microbiology and Immunology, University of Missouri, Columbia, Missouri, United States of America; 3 Department of Biomedical Sciences, College of Veterinary Medicine, University of Missouri, Columbia, Missouri, United States of America; 4 Department of Neurology, School of Medicine, University of Missouri, Columbia, Missouri, United States of America; 5 Department of Biomedical, Biological & Chemical Engineering, College of Engineering, University of Missouri, Columbia, Missouri, United States of America; 6 Department of Molecular and Integrative Physiology, University of Michigan, Ann Arbor, Michigan, United States of America; 7 Institute of Gerontology, University of Michigan, Ann Arbor, Michigan, United States of America; Bangor University, UNITED KINGDOM

## Abstract

Some acute exercise effects are influenced by postexercise (PEX) diet, and these diet-effects are attributed to differential glycogen resynthesis. However, this idea is challenging to test rigorously. Therefore, we devised a novel genetic model to modify muscle glycogen synthase 1 (GS1) expression in rat skeletal muscle with an adeno-associated virus (AAV) short hairpin RNA knockdown vector targeting GS1 (shRNA-GS1). Contralateral muscles were injected with scrambled shRNA (shRNA-Scr). Muscles from exercised (2-hour-swim) and time-matched sedentary (Sed) rats were collected immediately postexercise (IPEX), 5-hours-PEX (5hPEX), or 9-hours-PEX (9hPEX). Rats in 5hPEX and 9hPEX experiments were refed (RF) or not-refed (NRF) chow. Muscles were analyzed for glycogen, abundance of metabolic proteins (pyruvate dehydrogenase kinase 4, PDK4; peroxisome proliferator-activated receptor γ coactivator-1α, PGC1α; hexokinase II, HKII; glucose transporter 4, GLUT4), AMP-activated protein kinase phosphorylation (pAMPK), and glycogen metabolism-related enzymes (glycogen phosphorylase, PYGM; glycogen debranching enzyme, AGL; glycogen branching enzyme, GBE1). shRNA-GS1 versus paired shRNA-Scr muscles had markedly lower GS1 abundance. IPEX versus Sed rats had lower glycogen and greater pAMPK, and neither of these IPEX-values differed for shRNA-GS1 versus paired shRNA-Scr muscles. IPEX versus Sed groups did not differ for abundance of metabolic proteins, regardless of GS1 knockdown. Glycogen in RF-rats was lower for shRNA-GS1 versus paired shRNA-Scr muscles at both 5hPEX and 9hPEX. HKII protein abundance was greater for 5hPEX versus Sed groups, regardless of GS1 knockdown or diet, and despite differing glycogen levels. At 9hPEX, shRNA-GS1 versus paired shRNA-Scr muscles had greater PDK4 and PGC1α abundance within each diet group. However, the magnitude of PDK4 or PGC1α changes was similar in each diet group regardless of GS1 knockdown although glycogen differed between paired muscles only in RF-rats. In summary, we established a novel genetic approach to investigate the relationship between muscle glycogen and other exercise effects. Our results suggest that exercise-effects on abundance of several metabolic proteins did not uniformly correspond to differences in postexercise glycogen.

## Introduction

One exercise session has multiple metabolic consequences in the recruited skeletal muscle [1–5]. Some of these outcomes are influenced by the postexercise diet [4, 6–8]. It would be valuable to identify the underlying mechanisms for these postexercise diet effects. Skeletal muscle glycogen concentration, which is highly responsive to exercise and postexercise carbohydrate intake, is proposed to influence these outcomes [9]. Because altered skeletal muscle glycogen concentration is only one of many consequences of exercise and diet, glycogen’s specific role is challenging to discern.

Glycogen synthase 1 (GS1) is the rate-limiting enzyme for glycogen synthesis in skeletal muscle [10, 11]. Therefore, altering skeletal muscle GS1 expression is a reasonable strategy to alter muscle glycogen concentration. The primary aim was to test the feasibility of a novel genetic approach to reduce GS1 protein abundance in rat skeletal muscle. To minimize the confounding effect of glycogen manipulation methods using exercise and refeeding, one muscle from the paired muscles of rats was injected with adeno-associated virus (AAV) small-hairpin RNA (shRNA) that targets GS1 (AAV-shRNA-GS1), and the contralateral muscle was injected with AAV-shRNA-Scrambled (Scr) to serve as the control. A secondary aim was to test if our genetic approach would successfully delay muscle glycogen resynthesis with postexercise refeeding.

Pending validation of our novel genetic model, we planned to apply this approach to gain new insights into the extent to which muscle glycogen concentration influences postexercise effects on the abundance of key metabolic proteins in skeletal muscle with or without subsequent refeeding. Pilegaard et al. [2] compared the effects of consuming a high carbohydrate versus a low carbohydrate diet at times ranging from 2 to 24 hours after an acute bout of exercise on the mRNA expression of metabolic genes in human skeletal muscle. The expression of several genes was greater during the postexercise period compared to pre-exercise, including pyruvate dehydrogenase kinase 4 (PDK4), peroxisome proliferator-activated receptor γ coactivator-1α (PGC1α), and hexokinase II (HKII). Consuming a low carbohydrate diet versus a high carbohydrate diet during recovery resulted in longer lasting postexercise elevation of the expression of PDK4 and PGC1α. Muscle glycogen was significantly resynthesized only in the high carbohydrate diet trial. Muscle glycogen restoration during postexercise recovery has been proposed to be a key determinant of postexercise changes in muscle gene expression. Cluberton et al. [8] measured the expression of multiple metabolic genes in skeletal muscle of men who performed an acute bout of cycling exercise on two occasions that differed only by their consumption of either a carbohydrate-free placebo beverage or a carbohydrate beverage during the 3 hours of the recovery phase. Muscle PDK4 expression was elevated above at rest and 3 hours after exercise in the placebo trial, but not in the carbohydrate trial. Muscle PGC1α measured at 3 hours postexercise was increased to a similar extent above resting values in both trials. Acute exercise increased the skeletal muscle glucose transporter type 4 (GLUT4) protein abundance, and this effect was enhanced by postexercise carbohydrate supplementation [6]. Our third aim was to use our novel genetic model to analyze exercise and diet effects on several functionally important proteins (PGC1α, GLUT4, HK II, and PDK4).

AMPK-activated protein kinase (AMPK) is an enzyme that regulates skeletal muscle mRNA and protein expression [12]. AMPK influences multiple metabolic processes including the expression and regulation of the transcriptional coactivator and regulator of energy metabolism PGC1α, expression of key mitochondrial proteins, glucose uptake, glycogen metabolism, lipid metabolism, and protein synthesis [12, 13]. An acute bout of exercise can induce AMPK phosphorylation and activity [13]. In addition, multiple studies have suggested that pre-exercise or postexercise carbohydrate diets can attenuate the acute exercise-induced changes in AMPK activity [14–16]. Our fourth aim was to evaluate the influence of our intervention on the activation of AMPK.

In addition to GS1, skeletal muscle glycogen can be regulated by other enzymes. Glycogen phosphorylase (PYGM), which catalyzes glycogen degradation by the lysis of a terminal α-1, 4-glycosidic bond and the release of glucose-1-phosphate from the glycogen polymer, is the rate-limiting enzyme for muscle glycogenolysis [17, 18]. Glycogen is a branched polymer of glucose that consists of multiple linear chains of glucose that are linked by α-1, 4-glycosidic bonds. Glycogen has regular branch points (approximately every 12 glucose units) that are the result of α-1,6-bonds created by the reaction catalyzed by the glycogen branching enzyme (GBE1) [19]. The lysis of α-1,6-bonds of glycogen is catalyzed by glycogen debranching enzyme (AGL) [20, 21]. Our final aim was to determine if reducing GS1 abundance influences the abundance of these proteins that regulate glycogen metabolism (PYGM, AGL, and GBE1).

## Materials and methods

### Materials

Chemicals were obtained from Sigma-Aldrich (St. Louis, MO) or Fisher Scientific (Hanover Park, IL) unless otherwise noted. The reagents and apparatus for SDS-PAGE and nonfat dry milk (no. 170–6404) were from Bio-Rad (Hercules, CA). Pierce MemCode Reversible Protein Stain Kit (#24585), bicinchoninic acid protein assay (#23225), Tissue Protein Extraction Reagent (T-PER; #78510). Anti-glycogen synthase I (GS1; #3893), anti-hexokinase II (HKII; #2867), Anti-phospho AMPKα Thr^172^ (pAMPKα^Thr172^; #50081, which recognizes phosphorylation on both α1 and α2 isoforms), anti-AMPK-α (AMPKα; #5831, which recognizes both α1 and α2 isoforms), anti-acetyl CoA carboxylase (ACC; #3676), anti-phospho ACC^Ser79/212^ (pACC^Ser79/212^; #3661), anti-TBC1D1 (TBC1D1; #4629), and anti-rabbit IgG horseradish peroxidase (HRP) conjugate (#7074) were from Cell Signaling Technology (Danvers, MA). Anti-glycogen branching enzyme 1(GBE1; #20313-1-AP), anti-glycogen debranching enzyme (AGL; #16582-1-AP), and anti-pyruvate dehydrogenase kinase 4 (PDK4; #12949-1-AP) was from Proteintech (Rosemont, IL). Anti-glucose transporter type 4 (GLUT4; #CBL243), anti-phospho TBC1D1 Ser^237^ (pTBC1D1 Ser^237^; #07–2268), anti-peroxisome proliferator-activated receptor co-activator-γ-1α (PGC1α; 516557), and enhanced chemiluminescence Luminata Forte Western HRP Substrate (#WBLUF0100) were from EMD Millipore (Billerica, MA). Anti-glycogen phosphorylase (PYGM; ab231963) was from Abcam (Boston, MA).

### Animal treatment

Animal studies were conducted in accordance with the guidelines from the Guide for the Care and Use of Laboratory Animals of the National Institutes of Health and with the approval of the University of Michigan Committee on Use and Care of Animals. Male Wistar rats (Charles River Laboratories, Wilmington, MA) were 9 to 10-week-old when muscle samples were collected.

### Preparation of AAV expressing short-hairpin RNAs

Potential target sequences (Glycogen synthase 1, GS1) were initially identified using predesigned shRNA database (MilliporeSigma). Candidate sequences were initially tested for efficacy of knocking down endogenous GS1 protein level by immunoblot in L6 myocytes by transient transfection of siRNA (S1 Fig). Briefly, L6 myocytes were seeded the day before transfection. L6 myocytes were incubated for 48 hours with 10 nM candidate siRNA that mixed in the OptiMEM with lipofectamine RNAiMax. The myocytes were harvested and analyzed for GS1 expression. Based on the L6 myocyte results, the target sequence (5´-CCTGGACTTCAACCTAGACAA-3´) was selected and annealed to short-hairpin RNA (shRNA) oligo (5´-CCTGGACTTCAACCTAGACAActcgagTTGTCTAGGTTGAAGTCCAGGttttt-3´), shRNA sequence finally was ligated to pCWB U6-CMV-eGFP, an adeno-associated virus (AAV) vector *cis*-plasmid, by XbaI and SalI sites. A scrambled shRNA sequence (5´-CGCGATAGCGCTAATAATTTC-3´) was also cloned to the pCWB U6-CMV-eGFP AAV vector *cis*-plasmid to be used as a control. The *cis*-plasmids were used in conventional triple plasmid transfection for the production of the AAV9 serotype vector. AAV9 vectors were purified through two rounds of CsCl ultracentrifugation and the titer was determined by quantitative PCR.

### AAV administration

AAV was administered to the epitrochlearis muscle of 6 to 7-week-old rats as previously described [22]. Briefly, the rats were anesthetized (2.5% isoflurane/100% oxygen), their forelimbs were shaved, and analgesic (5 mg/kg carprofen) was subcutaneously injected. A 5- to 7-mm skin incision was made, and the exposed epitrochlearis was rinsed with sterile phosphate buffered saline (PBS). One epitrochlearis muscle of each rat was injected with GS1-targeting shRNA AAV (shRNA-GS1; 1.75X10^11^ vg/muscle). The contralateral muscle was injected with scramble shRNA AAV (shRNA-Scr; 1.75X10^11^ vg/muscle). The incision was sutured. Terminal experiments were performed at 3 to 4 weeks post-injection.

**Fig 1:**
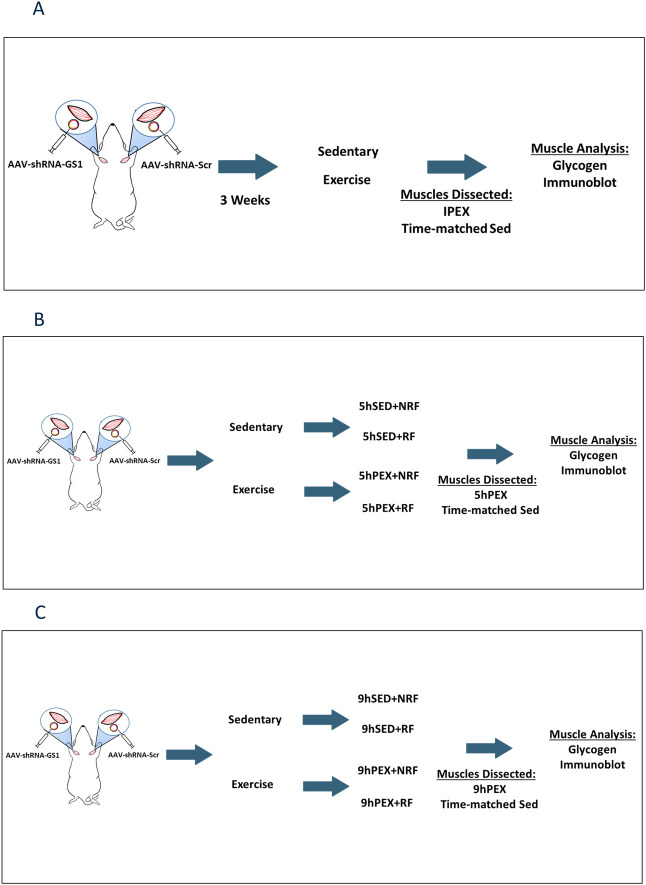
The experimental design for each time point. (A) IPEX and time-matched sedentary. (B) 5hPEX and time-matched sedentary with and without refeeding. (C) 9hPEX and time-matched sedentary with and without refeeding. The figure was created with BioRender.com.

Rats were fed rodent chow (Laboratory Diet no. 5L0D; LabDiet, St. Louis, MO) until fasted (1700 h the day before the experiment). The following day at ~0900, rats either swam or remained sedentary. The exercise protocol was swimming in a barrel filled with water (35°C, 45 cm depth, 6 rats swimming at a time) for four 30-min bouts with 5-min rest between bouts [22]. Then exercised rats along with time-matched, sedentary controls were anesthetized with an intraperitoneal injection of ketamine-xylazine cocktail (50 mg/kg ketamine and 5 mg/kg xylazine). Three experiments were performed to evaluate the key outcomes at key timepoints: immediately postexercise (IPEX), 5 hr postexercise (5hPEX), and 9 hr postexercise (9hPEX) along with time-matched sedentary. The 5hPEX and 9hPEX groups along with time-matched sedentary groups were either refed (RF) or not refed (NRF; provided ad libitum access to rodent chow) postexercise. The experimental design for each timepoint is depicted in Fig 1A (IPEX), 1B (5hPEX), and 1C (9hPEX). Both RF and NRF groups were also provided ad libitum access to water. In total, there were 10 groups of rats (with either shRNA-GS1 or shRNA-scr injected into the paired muscles from each rat, and thus 20 conditions): IPEX time-matched Sed (0hSed), IPEX, 5hPEX with refeeding (5hPEX-RF), 5hPEX without refeeding (5hPEX-NRF), 5hPEX time-matched Sed with refeeding (5hSed-RF), and without refeeding (5hSed-NRF), 9hPEX with refeeding (9hPEX-RF), 9hPEX without refeeding (9hPEX-NRF), 9hPEX time-matched Sed with refeeding (9hSed-RF), without refeeding (9hSed-NRF).

### Muscle lysate preparation

Frozen muscles were bisected. One portion used for immunoblotting, and the other for glycogen measurement. The muscle portions used for glycogen measurement were weighed and then homogenized in ice-cold water with a glass pestle attached to a motorized homogenizer (Caframo, Georgian Bluffs, ON, Canada). These homogenates were heated for 5 min at 95°C to inactivate enzymes, and then centrifuged (13,000 g for 5 min). The supernatants were transferred to microfuge tubes and used for subsequent glycogen analysis as described below.

The muscle portions used for immunoblotting were weighed and then homogenized with the motorized homogenizer described above in ice-cold lysis buffer (T-PER supplemented with 1 mM EDTA, 1 mM EGTA, 2.5mM sodium pyrophosphate, 1 mM sodium orthovanadate, 1 mM β-glycerophosphate, 1 μg/ml leupeptin, and 1 mM phenylmethylsulfonyl fluoride). These lysates were rotated for 1h at 4°C before centrifugation (15,000 g for 15 min at 4°C). The supernatants were transferred to 1.5 ml microfuge tubes and used for subsequent immunoblotting as described below.

### Muscle glycogen measurement

Muscle glycogen level was determined using a Glycogen Assay Kit (#MAK016, Sigma-Aldrich, St. Louis, MO) according to the manufacturer’s protocol. Absorbance was measured at 570 nm with a microplate reader [23].

### Immunoblotting

For each sample, an equal amount of lysate protein was mixed with 6x Laemmli buffer. The mixed samples were subjected to SDS-PAGE, and transferred to polyvinylidene difluoride membranes. Equal loading was confirmed with the MemCode protein stain kit [23]. Membranes were blocked with TBST (Tris-buffered saline pH 7.5 with 0.1% of Tween-20) that was mixed with either 5% of nonfat milk or bovine serum albumin (BSA) for 1 hr at room temperature. Membranes were then washed (3 X 5 min with TBST), and incubated in appropriate primary antibodies (5% of nonfat milk or BSA, overnight at 4°C). Incubated membranes were washed (3 X 5 min with TBST), and incubated in secondary antibody (5% of nonfat milk or BSA, 1 hr at room temperature). Before membranes were subjected to enhanced chemiluminescence, they were washed (3 X 5 min with TBST, and 2 X 5 min with Tris-buffered saline pH 7.5, TBS), and then quantified by densitometry (AlphaView; ProteinSimple, San Jose, CA). Individual values were normalized to the mean value for all samples on the same membrane.

### Statistics

Multilevel mixed-effects linear regression analysis was used to compare group means with Stata/SE 17.0 statistical software (Stata Corporation). Multilevel mixed-effects linear regression analysis was performed because the results derived for paired muscles (injected with either shRNA-GS1 and shRNA-Scr) from the same rat are inherently correlated. A p-value ≤ 0.05 was considered statistically significant.

## Results

### IPEX: Immunoblotting and muscle glycogen

**Fig 2:**
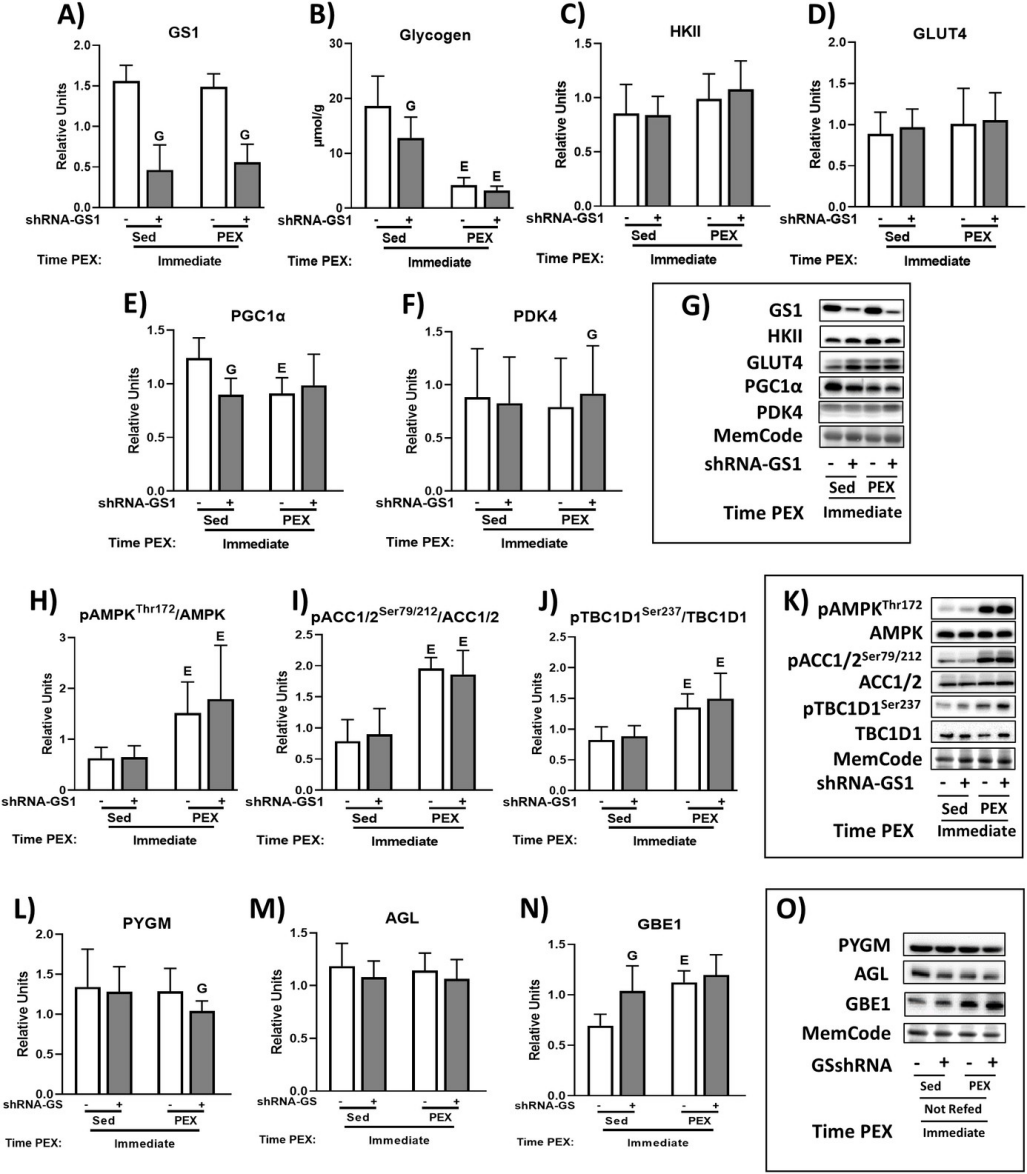
Protein abundance, glycogen concentration, and protein phosphorylation in skeletal muscle from immediately postexercised rats or time-matched sedentary control rats. (A) Glycogen synthase abundance. (B) Glycogen. (C) HKII abundance. (D) GLUT4 abundance. (E) PGC1α abundance. (F) PDK4 abundance. (G) Representative immunoblots. (H) pAMPK^Thr172^/AMPK ratio. (I) pACC1/2^Ser79/212^/ACC1/2 ratio. (J) pTBC1D1^Ser237^/ TBC1D1 ratio. (K) Representative immunoblots. (L) PYGM abundance. (M) AGL abundance. (N) GBE1 abundance. (O) Representative immunoblots. shRNA-GS1 versus shRNA-Scr within the same exercise group (IPEX or Sedentary), ^G^*P* < 0.05; IPEX versus Sedentary within the same AAV group (shRNA-GS1 or shRNA-Scr), ^E^*P* < 0.05. Comparisons between the two groups were analyzed by a multilevel mixed-effects linear regression analysis. Values are means ± SD; n = 6/group for all the immunoblots and glycogen.

GS1 protein abundance was significantly lower for shRNA-GS1 versus shRNA-Scr muscles (Fig 2A). Among shRNA-Scr muscles, glycogen level was lower in IPEX versus 0hSed (Fig 2B). Among the shRNA-GS1 muscles, glycogen level was lower in IPEX versus 0hSed (Fig 2B). In 0hSed rats, glycogen level was lower in shRNA-GS1 versus shRNA-Scr (Fig 2B). There were no significant effects of IPEX or GS1 knockdown on HKII, GLUT4, and AGL (Fig 2C, 2D, and 2M). In 0hSed rats, PGC1α protein abundance was lower in shRNA-GS1 versus shRNA-Scr. Among shRNA-Scr muscles, PGC1α abundance was lower in IPEX versus 0hSed (Fig 2E). In IPEX rats, PDK4 abundance was greater in shRNA-GS1 versus shRNA-Scr (Fig 2F). The ratio of pAMPK^Thr172^/AMPK, pACC^Ser79/212^/ACC1/2, and pTBC1D1^Ser237^/TBC1D1 was greater for IPEX versus 0hSed regardless of GS1 knockdown (Fig 2H, 2I and 2J). In IPEX rats, PYGM abundance was lower in shRNA-GS1 versus shRNA-Scr (Fig 2L). In 0hSed rats, GBE1 abundance was greater in shRNA-GS1 versus shRNA-Scr (Fig 2N). Among shRNA-Scr muscles, GBE1 abundance was greater in IPEX versus 0hSed (Fig 2N).

### 5hPEX: Immunoblotting and muscle glycogen

**Fig 3:**
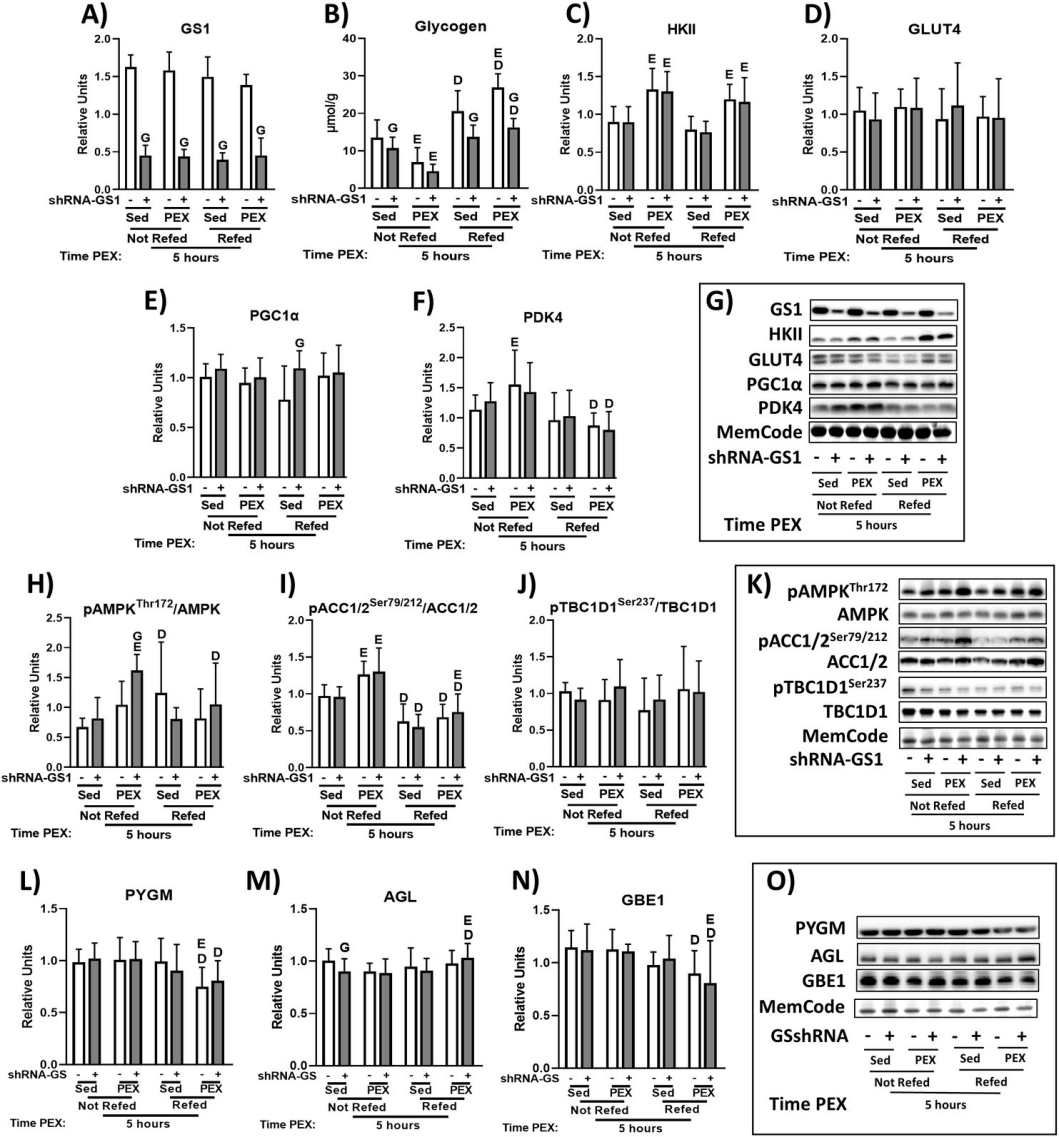
Protein abundance, glycogen concentration, and protein phosphorylation determined in skeletal muscle from 5-hour postexercised rats or time-matched sedentary control rats with or without refeeding. (A) Glycogen synthase abundance. (B) Glycogen. (C) HKII abundance. (D) GLUT4 abundance. (E) PGC1α abundance. (F) PDK4 abundance. (G) Representative immunoblots. (H) pAMPK^Thr172^/AMPK ratio. (I) pACC1/2^Ser79/212^/ACC1/2 ratio. (J) pTBC1D1^Ser237^/ TBC1D1 ratio. (K) Representative immunoblots. (L) PYGM abundance. (M) AGL abundance. (N) GBE1 abundance. (O) Representative immunoblots. shRNA-GS1 versus shRNA-Scr within the same exercise group (5hPEX or 5hSed) and diet group (refed or not refed), ^G^*P* < 0.05; 5hPEX versus 5hSed within the same AAV (shRNA-GS1 or shRNA-Scr) and diet group (refed or not refed), ^E^*P* < 0.05; 5h-RF versus 5h-NRF within the same AAV (shRNA-GS1 or shRNA-Scr) and exercise group (5hPEX or 5hSed), ^D^*P* < 0.05. Comparisons between the two groups were analyzed by a multilevel mixed-effects linear regression analysis. Values are means ± SD; n = 6-12/group for all the immunoblots and glycogen.

In the 5hSed-NRF, 5hPEX-NRF, 5hSed-RF, and 5hPEX-RF rats, GS1 abundance was significantly lower in shRNA-GS1 versus shRNA-Scr muscles (Fig 3A). In the 5h-NRF rats, glycogen level was lower in 5hPEX versus 5hSed regardless of GS1 knockdown (Fig 3B). In the 5hSed-NRF rats, glycogen level was lower in shRNA-GS1 versus shRNA-Scr muscles (Fig 3B). In the 5h-RF rats, glycogen level was lower in shRNA-GS1 versus shRNA-Scr regardless of exercise status (Fig 3B). In the shRNA-Scr muscles, glycogen level was greater for 5hPEX-RF versus 5hSed-RF (Fig 3B). In the 5hPEX rats, glycogen level was greater for 5h-RF versus 5h-NRF regardless of GS1 knockdown (Fig 3B). In the 5hSed rats, glycogen level in shRNA-Scr muscles were greater for 5h-RF versus 5h-NRF (Fig 3B). HKII protein abundance was significantly greater in 5hPEX versus 5hSed groups regardless of refeeding status or GS1 knockdown (Fig 3C). There were no significant effects of 5hPEX, diet, or GS1 knockdown on GLUT4 (Fig 3D). In the 5hSed-RF rats, the abundance of PGC1α was greater for shRNA-GS1 versus shRNA-Scr (Fig 3E). PDK4 protein abundance was lower for 5hPEX-RF versus 5hPEX-NRF irrespective of GS1 knockdown (Fig 3F). PDK4 abundance in shRNA-Scr muscles was greater for 5hPEX-NRF versus 5hSed-NRF (Fig 3F). In 5h-NRF rats, the pAMPK^Thr172^/AMPK ratio in shRNA-GS1 muscle was greater for 5hPEX versus 5hSed (Fig 3H). In 5hPEX-NRF rats, the pAMPK^Thr172^/AMPK ratio was greater for shRNA-GS1 versus shRNA-Scr (Fig 3H). In 5hSed rats, the pAMPK^Thr172^/AMPK ratio in shRNA-Scr muscle was greater for 5h-RF versus 5h-NRF (Fig 3H). In 5hPEX rats, the pAMPK^Thr172^/AMPK ratio in shRNA-GS1 muscle was lower for 5h-RF versus 5h-NRF (Fig 3H). In 5h-NRF rats, the ratio of pACC1/2^Ser79/212^/ACC1/2 was greater for 5hPEX versus 5hSed regardless of GS1 knockdown (Fig 3I). In 5hSed rats, the ratio of pACC1/2^Ser79/212^/ACC1/2 was lower for 5h-RF versus 5h-NRF groups regardless of GS1 knockdown (Fig 3I). In 5hPEX rats, the ratio of pACC1/2^Ser79/212^/ACC1/2 was lower for 5h-RF versus 5h-NRF groups regardless of GS1 knockdown (Fig 3I). In 5h-RF rats, the pACC1/2^Ser79/212^/ACC1/2 ratio in shRNA-GS1 muscle was greater for 5hPEX versus 5hSed (Fig 3I). PYGM and GBE1 abundance were lower for 5hPEX-RF versus 5hPEX-NRF irrespective of GS1 knockdown (Fig 3L and 3N). In the 5h-RF rats, the PYGM abundance in shRNA-Scr muscle was greater for 5hPEX versus 5hSed (Fig 3L). In the 5hSed-NRF rats, the abundance of AGL was lower for shRNA-GS1 versus shRNA-Scr (Fig 3M). In the 5hPEX rats, AGL abundance was greater in 5h-RF versus 5h-NRF (Fig 3M). In the 5h-RF rats, AGL abundance was greater in 5hPEX versus 5hSed (Fig 3M). In the 5h-RF rats, GBE1 abundance was lower in 5hPEX versus 5hSed (Fig 3N).

### 9hPEX: Immunoblotting and muscle glycogen

**Fig 4:**
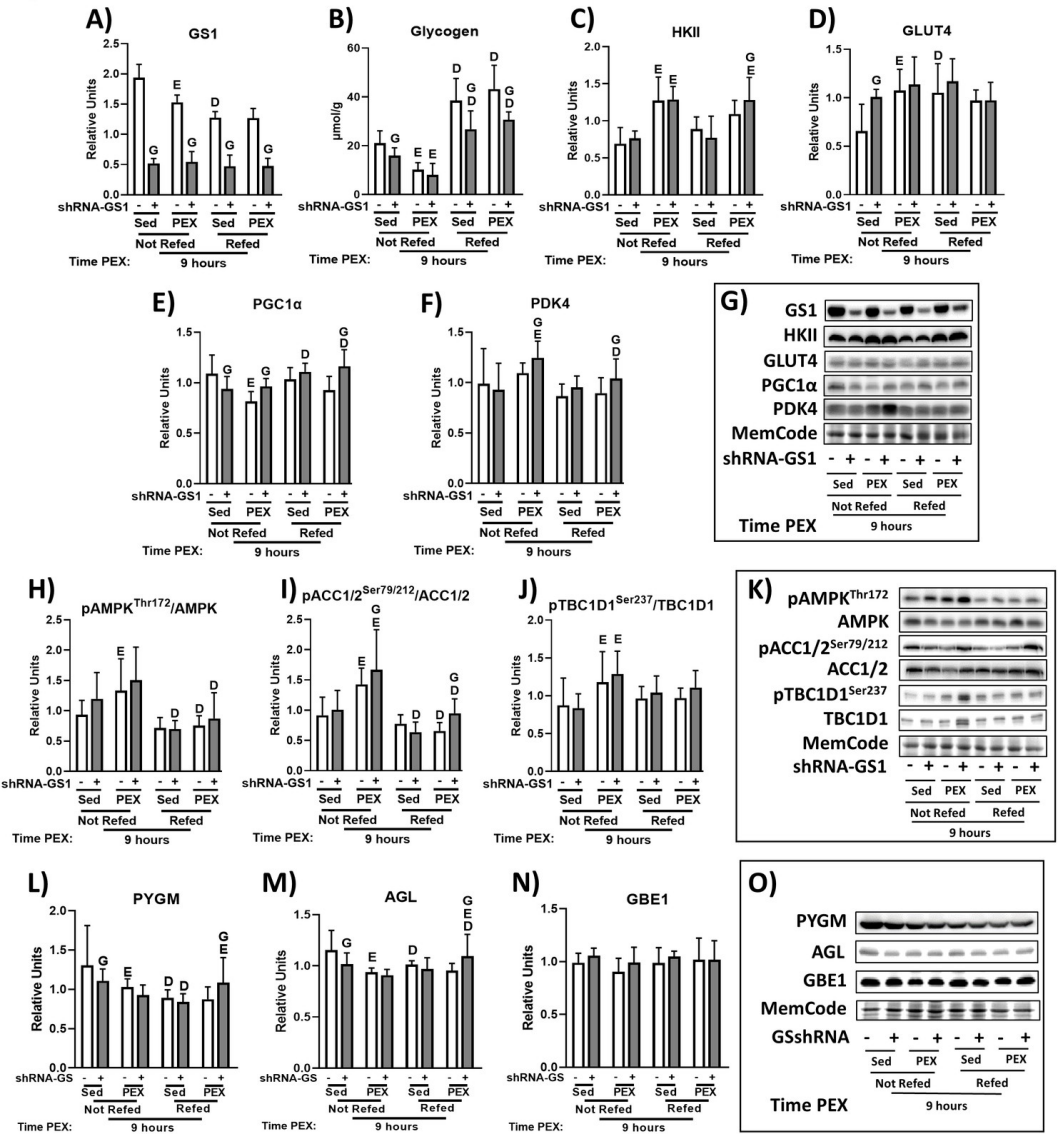
Protein abundance, glycogen concentration, and protein phosphorylation determined in skeletal muscle from 9-hour postexercised rats or time-matched sedentary control rats with or without refeeding. (A) Glycogen synthase abundance. (B) Glycogen. (C) HKII abundance. (D) GLUT4 abundance. (E) PGC1α abundance. (F) PDK4 abundance. (G) Representative immunoblots. (H) pAMPK^Thr172^/AMPK ratio. (I) pACC1/2^Ser79/212^/ACC1/2 ratio. (J) pTBC1D1^Ser237^/ TBC1D1 ratio. (K) Representative immunoblots. (L) PYGM abundance. (M) AGL abundance. (N) GBE1 abundance. (O) Representative immunoblots. shRNA-GS1 versus shRNA-Scr within the same exercise group (9hPEX or 9hSed) and diet group (refed or not refed, ^G^*P* < 0.05; 9hPEX versus 9hSed within the same AAV (shRNA-GS1 or shRNA-Scr) and diet group (refed or not refed), ^E^*P* < 0.05; 9h-RF versus 9h-NRF within the same AAV (shRNA-GS1 or shRNA-Scr) and exercise group (9hPEX or 9hSed), ^D^*P* < 0.05. Comparisons between the two groups were analyzed by a multilevel mixed-effects linear regression analysis. Values are means ± SD; n = 6/group for all the immunoblots and glycogen.

In the 9hSed-NRF, 9hPEX-NRF, 9hSed-RF, and 9hPEX-RF rats, GS1 abundance was significantly lower in shRNA-GS1 versus shRNA-Scr muscles (Fig 4A). In the 9h-NRF rats, glycogen level was lower in 9hPEX versus 9hSed regardless of GS1 knockdown (Fig 4B). In the 9hSed-NRF rats, glycogen level was lower in shRNA-GS1 versus shRNA-Scr muscles (Fig 4B). In the 9h-RF rats, glycogen level was lower in shRNA-GS1 versus shRNA-Scr regardless of exercise status (Fig 4B). In 9hSed rats, glycogen level was greater for 9h-RF versus 9h-NRF regardless of GS1 knockdown (Fig 4B). In 9hPEX rats, glycogen level was greater for 9h-RF versus 9h-NRF regardless of GS1 knockdown (Fig 4B). In the 9h-NRF rats, HKII abundance was greater for 9hPEX versus 9hSed regardless of GS1 knockdown (Fig 4C). In 9h-RF rats, HKII abundance in shRNA-GS1 muscle was greater for 9hPEX versus 9hSed (Fig 4C). In 9hPEX-RF rats, the abundance of HKII was greater for shRNA-GS1 versus shRNA-Scr (Fig 4C). In the 9hSed-NRF rats, GLUT4 abundance was greater for shRNA-GS1 versus shRNA-Scr (Fig 4D). In the 9h-NRF rats, GLUT4 abundance in shRNA-Scr muscle was greater for 9hPEX versus 9hSed (Fig 4D). In the 9hSed rats, GLUT4 abundance in shRNA-Scr muscle was greater for 9h-RF versus 9h-NRF rats (Fig 4D). In 9h-NRF rats, PGC1α abundance in shRNA-Scr muscle was lower for 9hPEX versus 9hSed (Fig 4E). In 9hSed-NRF rats, PGC1α abundance was lower for shRNA-GS1 versus shRNA-Scr muscle (Fig 4E). In 9hPEX-NRF rats, PGC1α abundance was greater for shRNA-GS1 versus shRNA-Scr muscle (Fig 4E). In 9hSed rats, PGC1α abundance in shRNA-GS1 muscle was greater for 9h-RF versus 9h-NRF (Fig 4E). In 9hPEX rats, PGC1α abundance in shRNA-GS1 muscle was greater for 9h-RF versus 9h-NRF (Fig 4E). In 9hPEX-RF rats, PGC1α abundance was greater for shRNA-GS1 versus shRNA-Scr (Fig 4E). In 9h-NRF rats, PDK4 abundance in shRNA-GS1 muscle was greater for 9hPEX versus 9hSed (Fig 4F). In 9hPEX-NRF rats, PDK4 abundance was greater for shRNA-GS1 versus shRNA-Scr (Fig 4F). In 9hPEX rats, PDK4 abundance in shRNA-GS1 muscle was lower for 9h-RF versus 9h-NRF (Fig 4F). In 9hPEX-RF rats, PDK4 abundance was greater for shRNA-GS1 versus shRNA-Scr (Fig 4F). In 9h-NRF rats, the pAMPK^Thr172^/AMPK ratio in shRNA-Scr muscle was greater for 9hPEX versus 9hSed (Fig 4H). In 9hSed rats, the pAMPK^Thr172^/AMPK ratio in shRNA-GS1 muscle was lower for 9h-RF versus 9h-NRF (Fig 4H). In 9hPEX rats, the pAMPK^Thr172^/AMPK ratio was lower for 9h-RF versus 9h-NRF regardless of GS1 knockdown (Fig 4H). The pACC1/2^Ser79/212^/ACC1/2 ratio was greater for 9hPEX versus 9hSed regardless of GS1 knockdown (Fig 4I). In 9hPEX-NRF rats, the pACC1/2^Ser79/212^/ACC1/2 ratio was greater for shRNA-GS1 versus shRNA-Scr muscle (Fig 4I). In 9hSed rats, the pACC1/2^Ser79/212^/ACC1/2 ratio in shRNA-GS1 muscle was lower for 9h-RF versus 9h-NRF (Fig 4I). In 9hPEX rats, the pACC1/2^Ser79/212^/ACC1/2 ratio was lower for 9h-RF versus 9h-NRF regardless of GS1 knockdown (Fig 4I). In 9hPEX-RF rats, the pACC1/2^Ser79/212^/ACC1/2 ratio was greater for shRNA-GS1 versus shRNA-Scr (Fig 4I). In 9h-NRF rats, the pTBC1D1^Ser237^/TBC1D1 ratio was greater for 9hPEX versus 9hSed regardless of GS1 knockdown (Fig 4J). In the 9hSed-NRF rats, PYGM and AGL abundance were lower in shRNA-GS1 versus shRNA-Scr (Fig 4L and 4M). In the 9h-NRF rats, PYGM and AGL abundance in shRNA-Scr muscles were lower for 9hPEX versus 9hSed (Fig 4L and 4M). In the 9h-Sed rats, PYGM abundance was lower for 9h-RF versus 9h-NRF regardless of GS1 knockdown (Fig 4L). In the 9hPEX-RF rats, PYGM and AGL abundance were greater for shRNA-GS1 versus shRNA-Scr (Fig 4L and 4M). In the 9h-RF rats, PYGM and AGL abundance in shRNA-GS1 muscle were greater for 9hPEX versus 9hSed (Fig 4L and 4M). In the 9hSed rats, AGL abundance in shRNA-Scr muscle was lower for 9h-RF versus 9h-NRF (Fig 4M). In the 9hPEX rats, AGL abundance in shRNA-GS1 muscle was greater for 9h-RF versus 9h-NRF (Fig 4M). There were no significant effects of 9hPEX, diet, or GS1 knockdown on GBE1 (Fig 4N).

## Discussion

The primary goal of this study was to create a novel genetic model that would induce a marked decrease in the GS1 protein abundance of a rat skeletal muscle but not in the contralateral muscle of the same rat. Our motivation was to enable unique comparisons between paired muscles that differed with regard to postexercise muscle glycogen concentration. The model successfully induced a substantial reduction in GS1 protein abundance. As anticipated, in rats that were refed after exercise, glycogen accumulation was substantially reduced in muscles with lower GS1 abundance compared to paired control muscles with normal GS1 abundance. The results of this study provided novel insights into the putative role of muscle glycogen accumulation in the effects of postexercise, carbohydrate ingestion on the abundance of important metabolic proteins.

Several aspects of the experimental design are notable. Firstly, analysis of paired muscles that differed for GS1 abundance was valuable because the contralateral muscles from each rat were exposed to identical concentrations of potential systemic, regulatory factors. Secondly, using partial GS1 knockdown, rather than the complete elimination of GS1 enabled more physiologically relevant comparisons. Finally, the postexercise timepoints for muscle analysis (IPEX, 5hPEX, and 9hPEX) were selected in the context of the results of earlier research. The greatest decrements in muscle glycogen and increases in AMPK activation (which are commonly used as indicators of the extent of metabolic challenge in response to acute exercise) are evident when evaluated IPEX [24–26]. Substantial muscle glycogen resynthesis can occur in rats that ingest a high carbohydrate diet during the initial 3 to 5 hours postexercise [27–29]. Elevation in the abundance of multiple, metabolic proteins in rat skeletal muscle can be detected at 5 to 10 hours postexercise [30–32].

The substantial decrement in GS1 protein abundance in shRNA-GS1-treated muscles resulted in moderately lower glycogen in sedentary rats. Importantly, glycogen in paired muscles from IPEX rats was reduced to similarly low concentrations, regardless of GS1 abundance. Having a comparable glycogen level in the paired muscles at this crucial time point prior to refeeding facilitated the interpretation of the comparison of the extent of glycogen accumulation between paired muscles in the subsequent refeeding experiments.

No significant IPEX-related increases in the abundance of the key metabolic proteins evaluated were detected with or without GS1 knockdown. Consistent with our finding of no IPEX-increase in HKII protein abundance, earlier studies found no significant increase in maximal hexokinase activity of muscle IPEX [33, 34]. It should also be noted that acute exercise can rapidly elevate HKII mRNA in rat muscle, with an increase detectable IPEX [35]. The absence of an IPEX-increase in muscle GLUT4 protein corresponded with the results for rats reported in previous research by Kuo et al. [36]. However, a subsequent study by the same researchers detected greater GLUT4 protein in rat muscle immediately after the completion of 6 hours of swim exercise [7]. Consistent with our results, several studies have reported no IPEX-effect on PGC1α protein abundance [37–39], but increased PGC1α protein in muscle has also been reported IPEX [40]. In contrast to the lack of an IPEX-effect on PDK4 abundance in the current study, muscle PDK4 protein abundance increased in mice immediately after treadmill exercise [30]. Taken together, the absence of IPEX-induced increases in PDK4, HKII, PGC1α, and GLUT4 protein abundance in the current study aligns with the findings of a number of prior studies, but elevated PDK4, PGC1α, or GLUT4 protein in muscle has also been previously observed IPEX by others.

Consistent with earlier research using the same exercise protocol [41–43], phosphorylation of AMPK and phosphorylation of its substrates ACC and TBC1D1 were markedly elevated IPEX. There was no evidence of a genetic difference between paired muscles for any of the markers of AMPK stimulation. The similarity between GS1 knockdown (shRNA-GS1) and control (shRNA-Scr) in IPEX values for muscle glycogen concentration and multiple markers of AMPK activation confirms a robust and parallel metabolic challenge by the exercise protocol in each of the paired muscles.

As expected, the lower GS1 abundance in the shRNA-GS1-treated muscles substantially reduced the refeeding-related increase in muscle glycogen at 5hPEX. Consistent with earlier studies [44, 45], there was a significant increase in muscle HKII protein abundance after one bout of acute exercise in control muscles. However, the magnitude of this exercise effect on HKII was not altered by either diet or GS1 knockdown even though there were substantial differences in muscle glycogen concentration among the 5hPEX groups. GLUT4 protein abundance was not altered by prior exercise, consistent with results for muscles studied 3 to 4 hours after the exercise protocol used in this study [41, 46]. PDK4 protein abundance in muscles without refeeding was greater only for shRNA-Scr muscles from 5hPEX versus sedentary rats. This result aligns with greater PDK4 protein abundance that was reported for human muscle at 6 hours postexercise [30], but not with an earlier study that found no change in muscle PDK4 protein abundance of mice at 3 hours after exercise [47]. There was no 5hPEX-related increase in muscle AMPK phosphorylation in the control muscles, which corresponds with earlier studies indicating a postexercise reversal of this outcome [42, 48]. However, AMPK phosphorylation in the shRNA-GS1 muscles from 5hPEX rats exceeded values for muscles from sedentary rats and for paired muscles with normal GS1 protein levels. ACC phosphorylation in rats that were not refed was greater in 5hPEX rats versus sedentary rats, regardless of GS1 knockdown. A small, but significant exercise effect on ACC phosphorylation was found only in the shRNA-GS1-treated muscles of refed rats. The results indicate that the largest 5hPEX effect on the abundance of the key proteins that were evaluated (greater HKII protein abundance), was unrelated to differences in either GS1 protein abundance or diet, in spite of markedly different glycogen levels between these conditions.

Several modest, but significant differences were detected between the paired muscles at 9hPEX. The muscles with lower GS1 abundance versus paired control muscles had a greater abundance of PDK4 and PGC1α within each diet group at this time point. It was notable that the magnitude of the GS1-related difference in abundance of either PDK4 or PGC1α was quite similar in each diet group, even though muscle glycogen differed between paired muscles only in the refed rats. These results argue against the differences between paired muscles for PDK4 and PGC1α at 9hPEX being the consequence of postexercise differences in muscle glycogen. Muscle ACC phosphorylation, often used as a surrogate for elevated AMPK activity, was greater in muscles with lower GS1 abundance at 9hPEX regardless of diet. In rats that were not refed, HKII abundance at 9hPEX exceeded the values for time-matched sedentary rats, regardless of GS1 knockdown. In the 9hPEX rats that were refed, muscles with lower GS1 abundance versus paired muscles had greater HKII abundance, lower glycogen concentration, and greater ACC phosphorylation. It is possible that differences in muscle glycogen at 9hPEX played a role in the greater HKII abundance in muscles with lower GS1 abundance at this time point. The only exercise-induced increases in GLUT4 protein abundance or AMPK phosphorylation at 9hPEX were in the shRNA-Scr muscle from rats that were not refed. The exercise effect on GLUT4 protein in the control muscle was consistent with earlier studies reporting that muscle GLUT4 protein can be increased at 5 to 16 h after acute exercise [6, 7, 49]. The effect on GLUT4 abundance in the 9hPEX rats was absent from shRNA-GS1 muscle, even though glycogen concentration was similarly low in the paired muscles from these rats. Taken together, the results are inconsistent with the idea that lower muscle glycogen at 9hPEX played a primary role in the exercise-related effects on metabolic protein abundance.

Three key enzymes that regulate glycogen metabolism (PYGM, AGL, and GBE1) were analyzed to evaluate if substantially reducing GS1 abundance would induce a modification in their expression in skeletal muscle. Without exception, in each of the exercise and diet conditions, the abundance of GS1 was dramatically lower in shRNA-GS1 versus shRNA-Scr muscles. In striking contrast, substantial and consistent differences between paired muscles were not observed for PYGM, AGL, or GBE1. In addition, no marked or uniform patterns of diet- or exercise-related changes in the abundance of these proteins were evident. The results for PYGM, AGL, and GBE1 do not reveal major compensatory alterations in the expression of these important, glycogen regulatory enzymes.

The most important outcome of this study was the development of a novel approach to investigate the relationship between muscle glycogen and exercise effects on key metabolic proteins. Another noteworthy finding was that although exercise-induced increases in the abundance of several metabolic proteins were detected, it was striking that the observed differences in postexercise muscle glycogen concentration did not consistently correspond to the enhanced protein abundance. Finally, this new genetic model has the potential to be useful in research focused on other consequences of exercise. For example, a similar approach could provide a unique perspective to explore the widely studied, but poorly understood relationship between muscle glycogen and postexercise insulin-stimulated glucose uptake.

## Supporting information

S1 Fig Validation of GS1 silencing using L6 cell. The target sequence for GS1 knockdown was identified through a predesigned shRNA database (MilliporeSigma). Either scrambled siRNA as a control or siRNA that targets GS1 was transfected into L6 cells for 48 hours. The abundance of GS1 and AKT was analyzed using a western blot. MemCode is an indicator of total protein and served as a loading control. siRNA that targeted GS1 effectively decreased the GS1 abundance in L6 cells without affecting Akt or total protein abundance.
(TIF)

S1 Raw images The blots used in the main figures are indicated using blue boxes. (PDF)

S1 File The values used to build graphs. Each table contains the data (Individual, mean, and standard deviation values) that were used to generate the graphs used in the main figures.
(XLSX)
