# Supplementary figures and images for: A novel genetic model provides a unique perspective on the relationship between postexercise glycogen concentration and increases in the abundance of key metabolic proteins after acute exercise
Source: PLoS One. 2024 Jan 30;19(1):e0295964. doi: 10.1371/journal.pone.0295964 (PMC10826964; doi:10.1371/journal.pone.0295964)

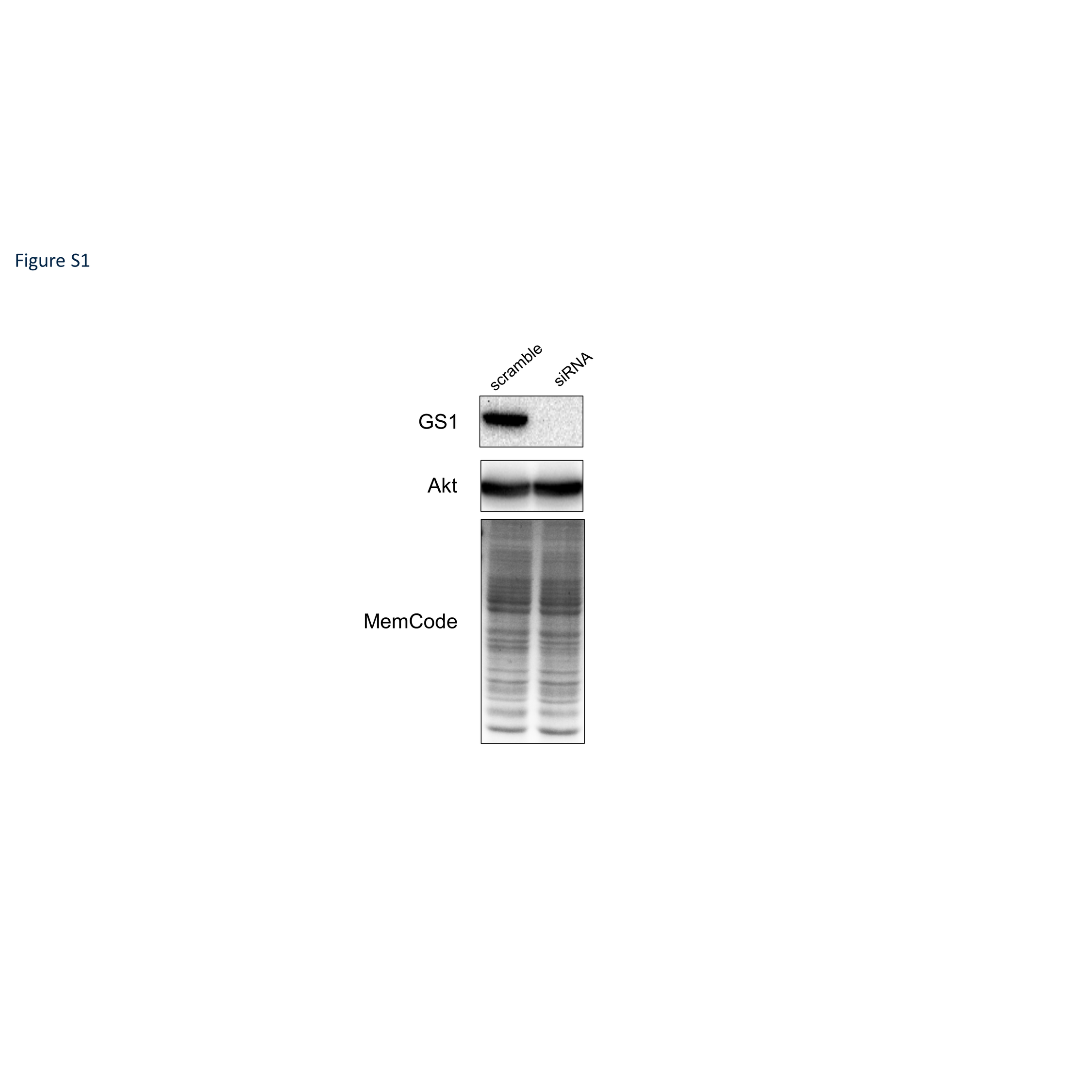

Supplement: S1 Fig — The target sequence for GS1 knockdown was identified through a predesigned shRNA database (MilliporeSigma). Either scrambled siRNA as a control or siRNA that targets GS1 was transfected into L6 cells for 48 hours. The abundance of GS1 and AKT was analyzed using a western blot. MemCode is an indicator of total protein and served as a loading control. siRNA that targeted GS1 effectively decreased the GS1 abundance in L6 cells without affecting Akt or total protein abundance. (TIF) [file pone.0295964.s001.tif]
