# Supplementary material for: A novel genetic model provides a unique perspective on the relationship between postexercise glycogen concentration and increases in the abundance of key metabolic proteins after acute exercise
Source: PLoS One. 2024 Jan 30;19(1):e0295964. doi: 10.1371/journal.pone.0295964 (PMC10826964; doi:10.1371/journal.pone.0295964)
Supplement: S1 Raw images — (PDF) [file pone.0295964.s002.pdf]

Figures 2&3 raw images

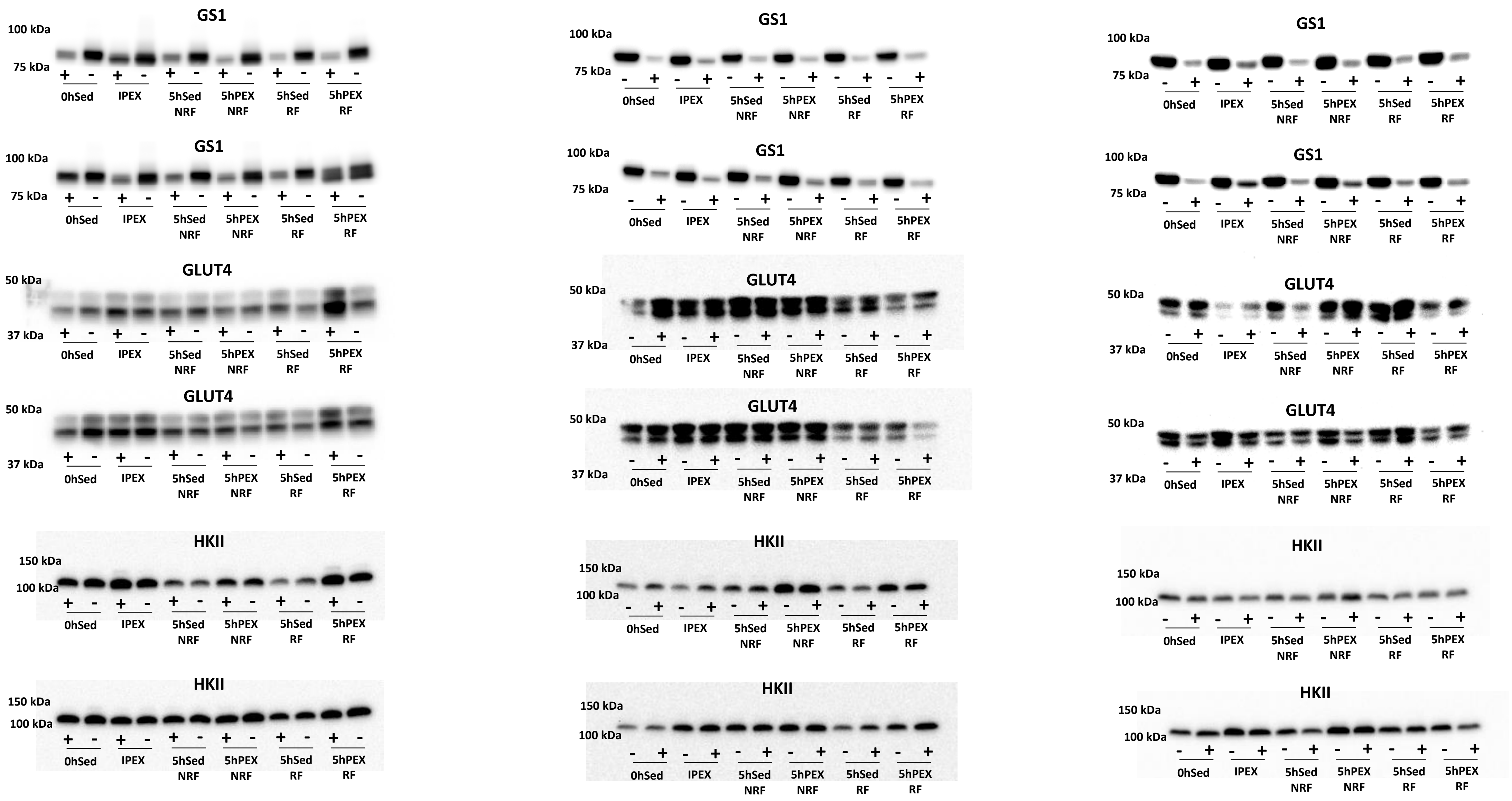

+:shRNA-GS1 injected muscle
-:shRNA-Scr injected muscle
All imiages were captured using the Chemiluminescence channel by FluorChemE by Proteinsimple

Figures 2&3 raw images

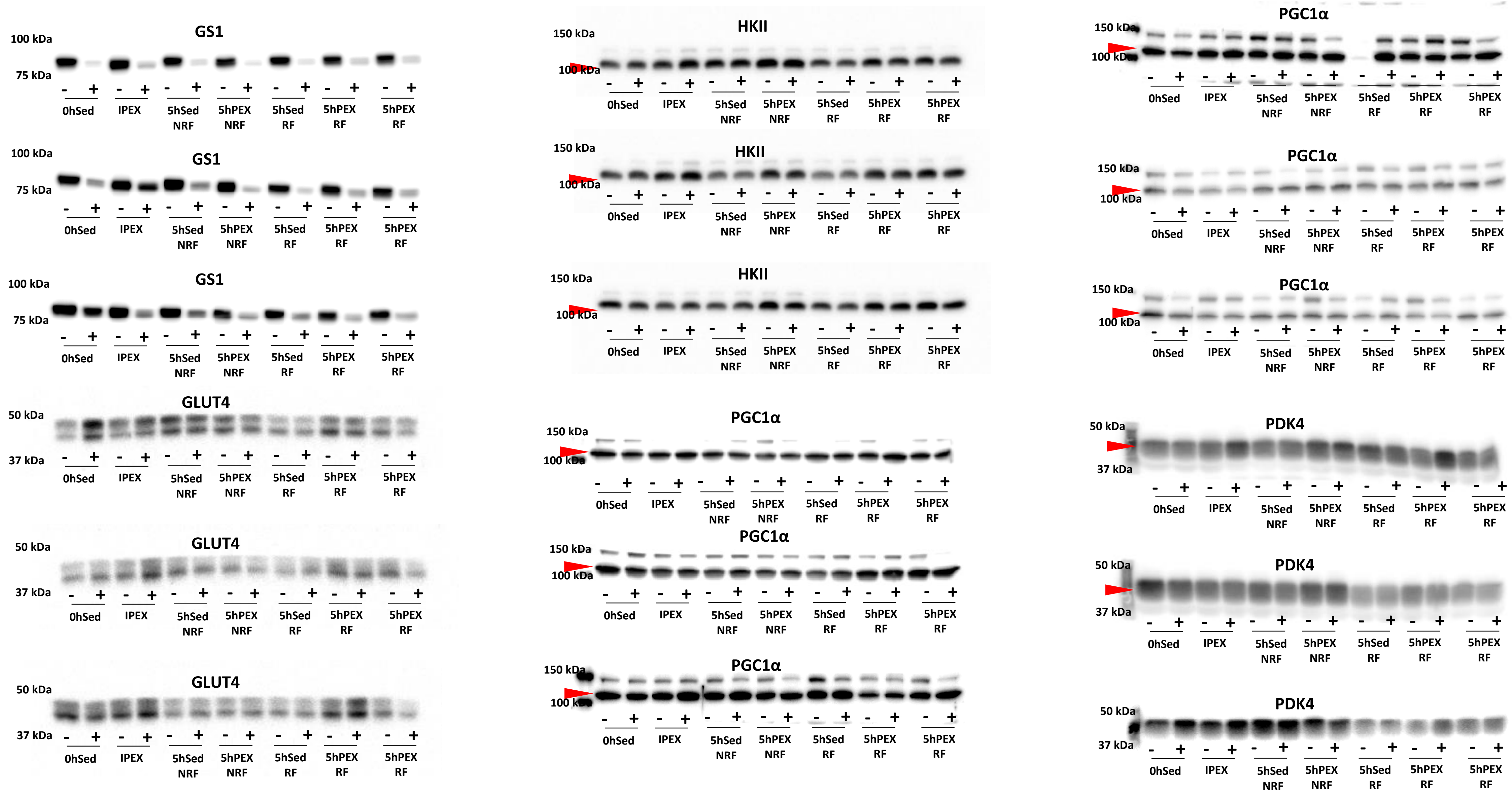

+:shRNA-GS1 injected muscle

-:shRNA-Scr injected muscle

All imiages were captured using the Chemiluminescence channel by FluorChemE by Proteinsimple

Denotes quantified band at expected molecular weight

Figures 2&3 raw images

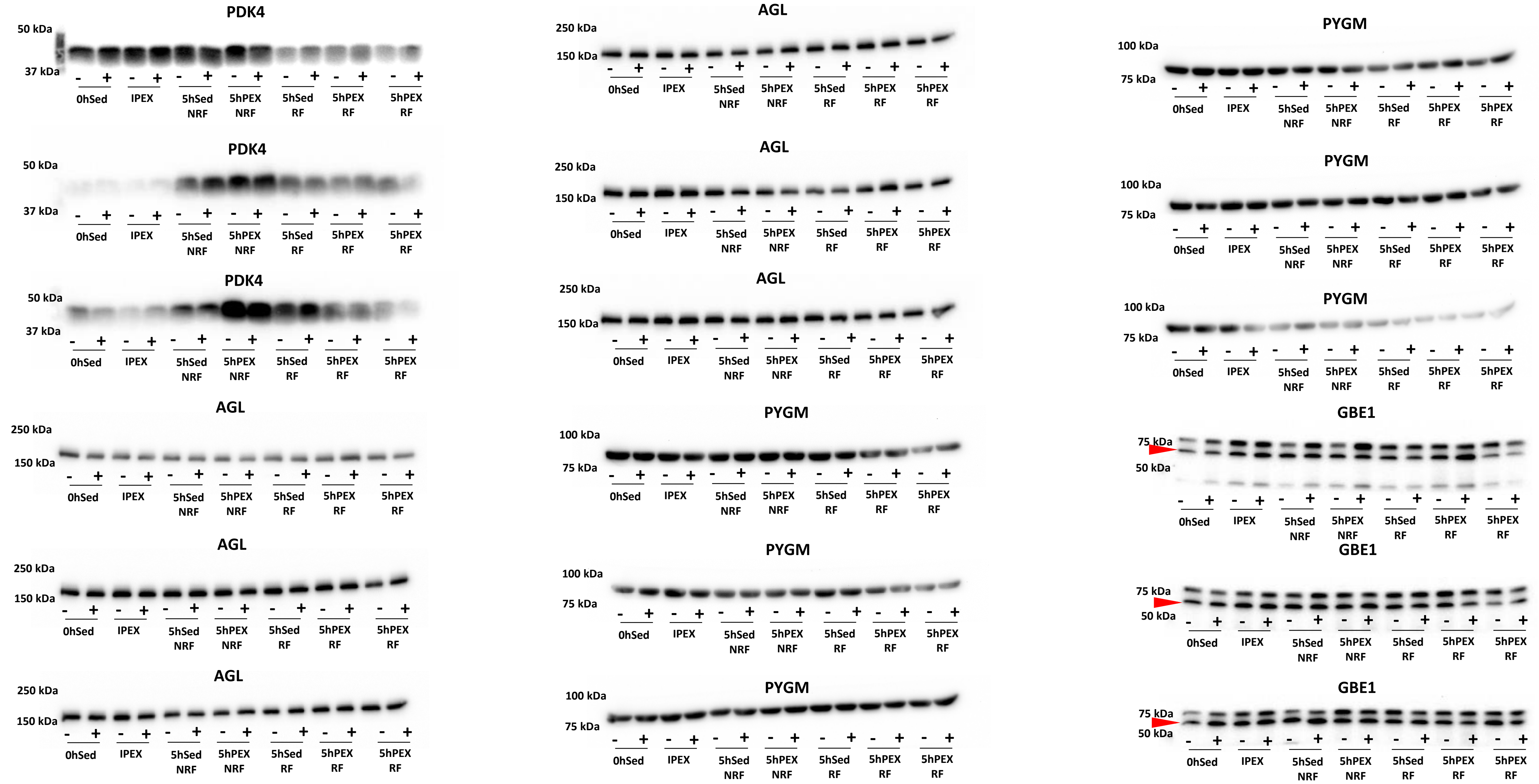

+:shRNA-GS1 injected muscle

-:shRNA-Scr injected muscle

All imiages were captured using the Chemiluminescence channel by FluorChemE by Proteinsimple

► Denotes quantified band at expected molecular weight

Figures 2&3 raw images

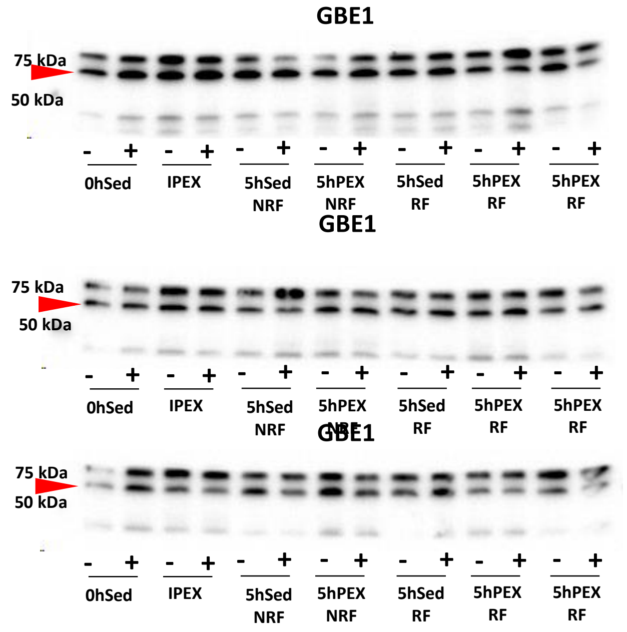

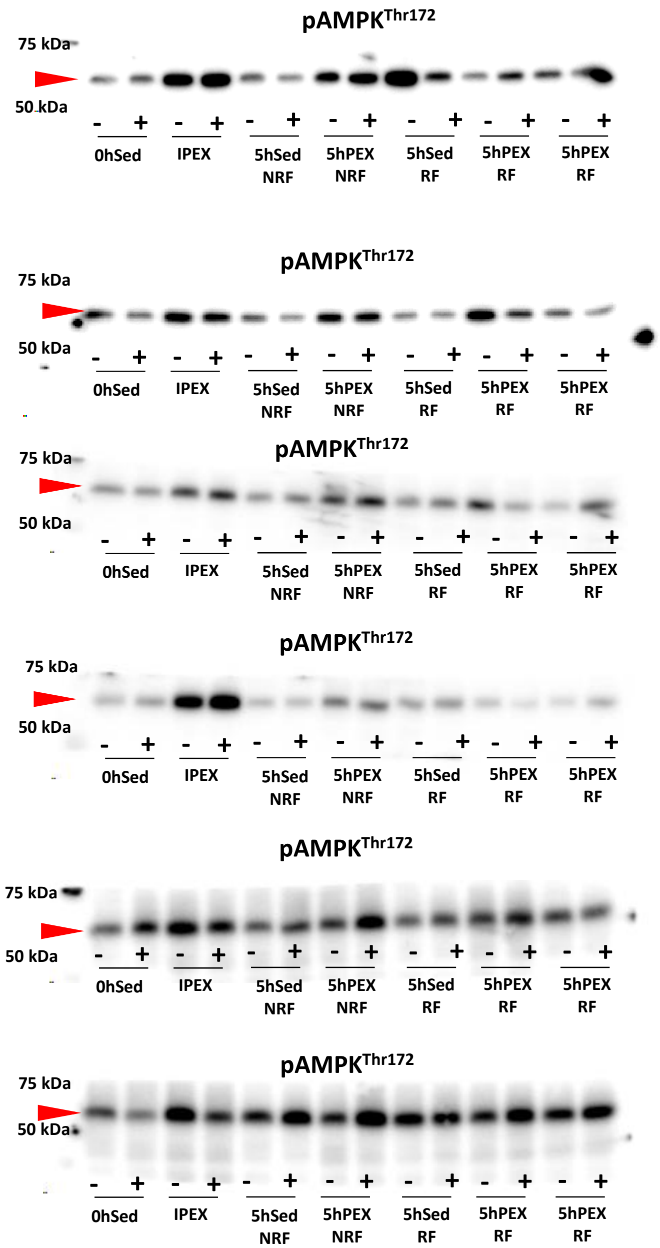

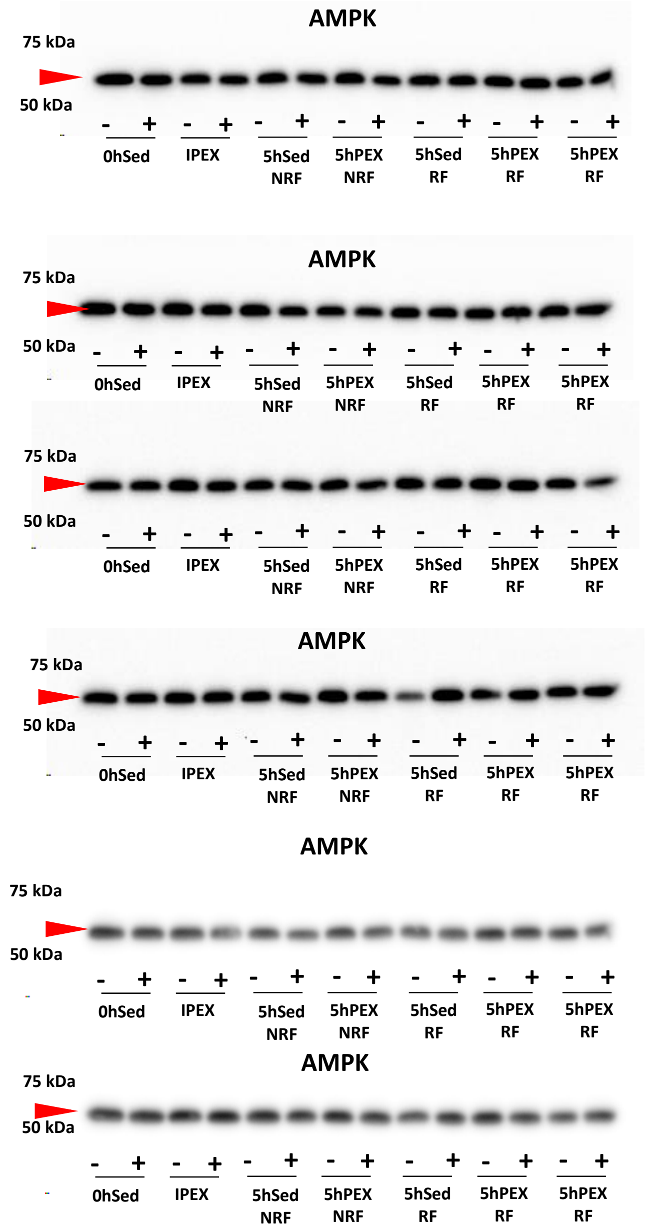

+:shRNA-GS1 injected muscle

-:shRNA-Scr injected muscle

All imiages were captured using the Chemiluminescence channel by FluorChemE by Proteinsimple

Denotes quantified band at expected molecular weight

Figures 2&3 raw images

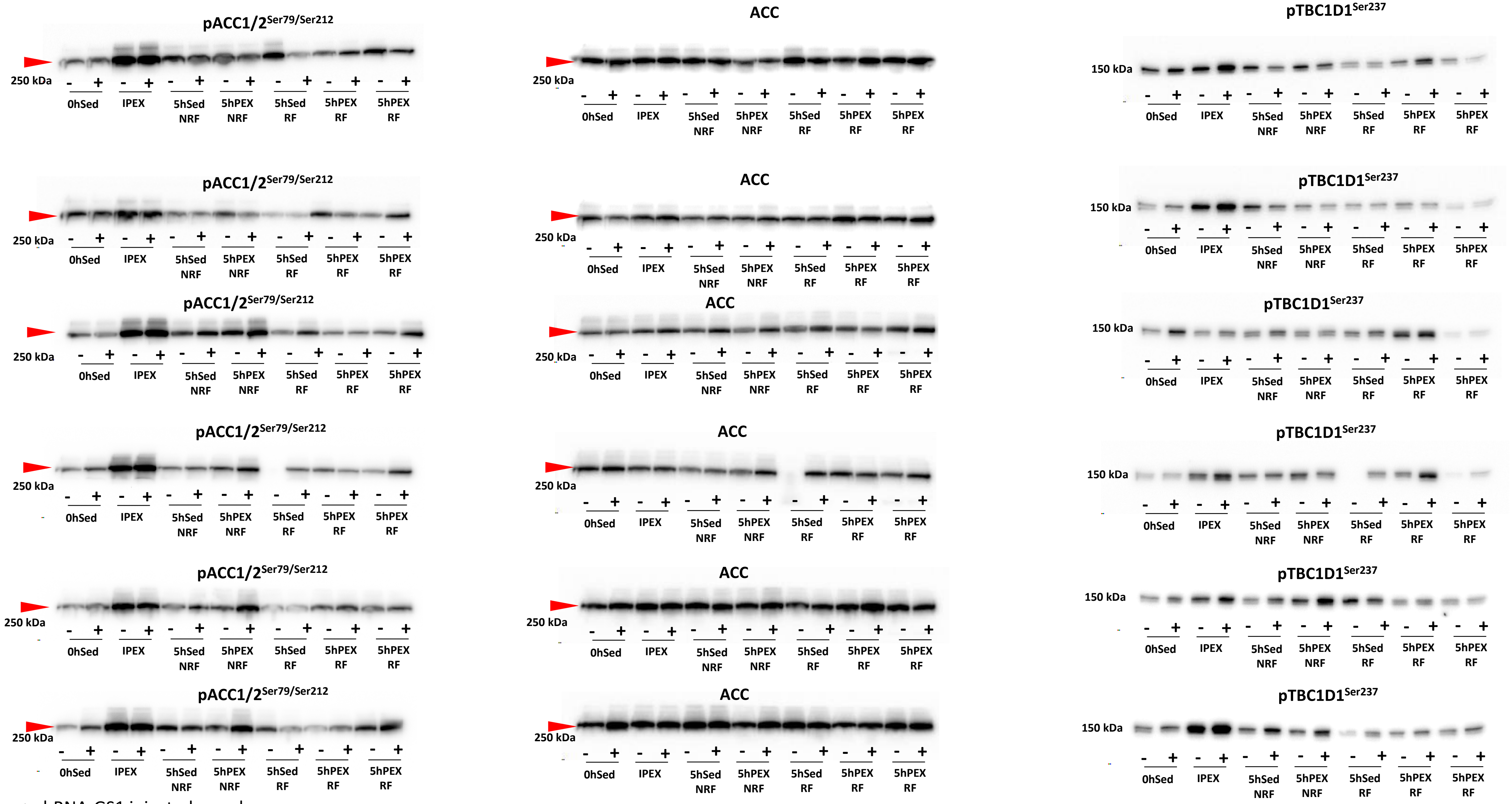

+:shRNA-GS1 injected muscle

-:shRNA-Scr injected muscle

All imiages were captured using the Chemiluminescence channel by FluorChemE by Proteinsimple

Denotes quantified band at expected molecular weight

Figures 2&3 raw images

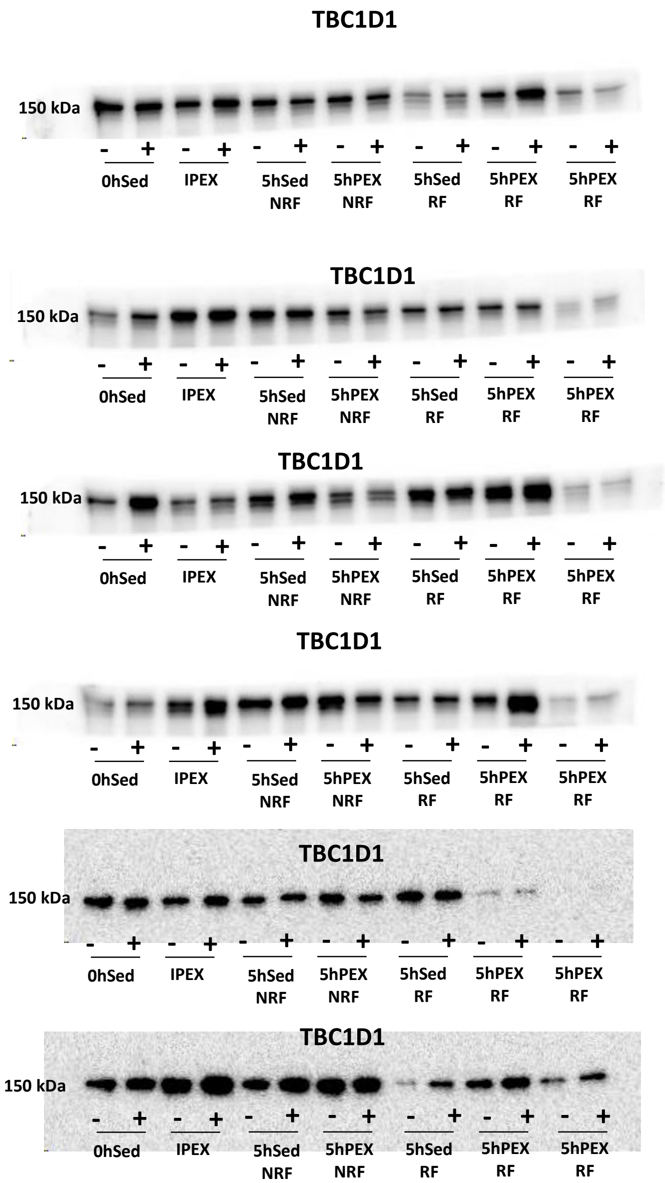

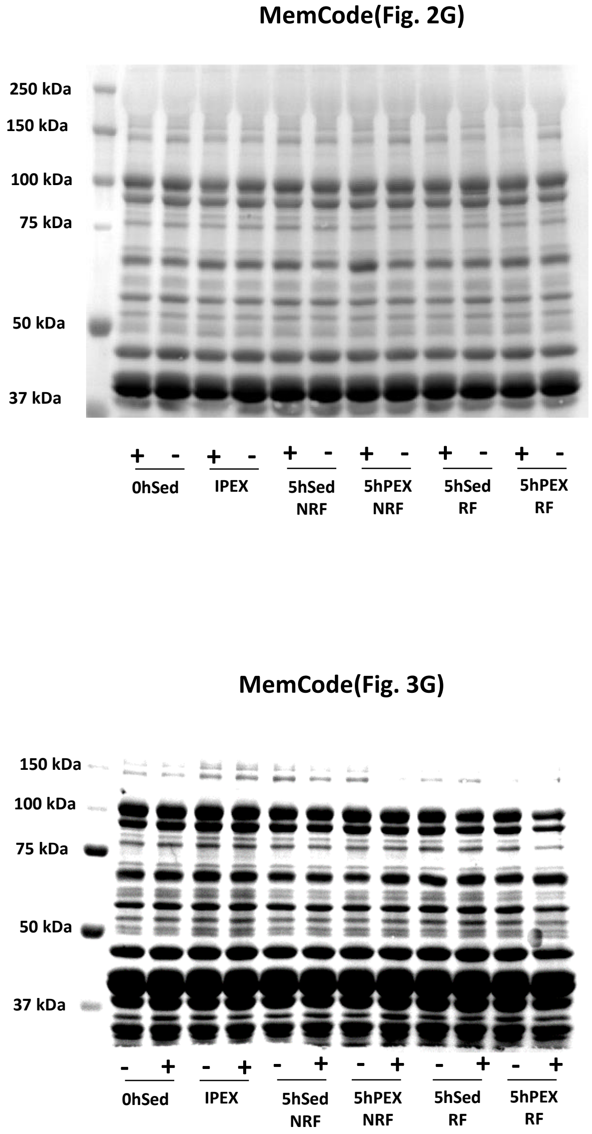

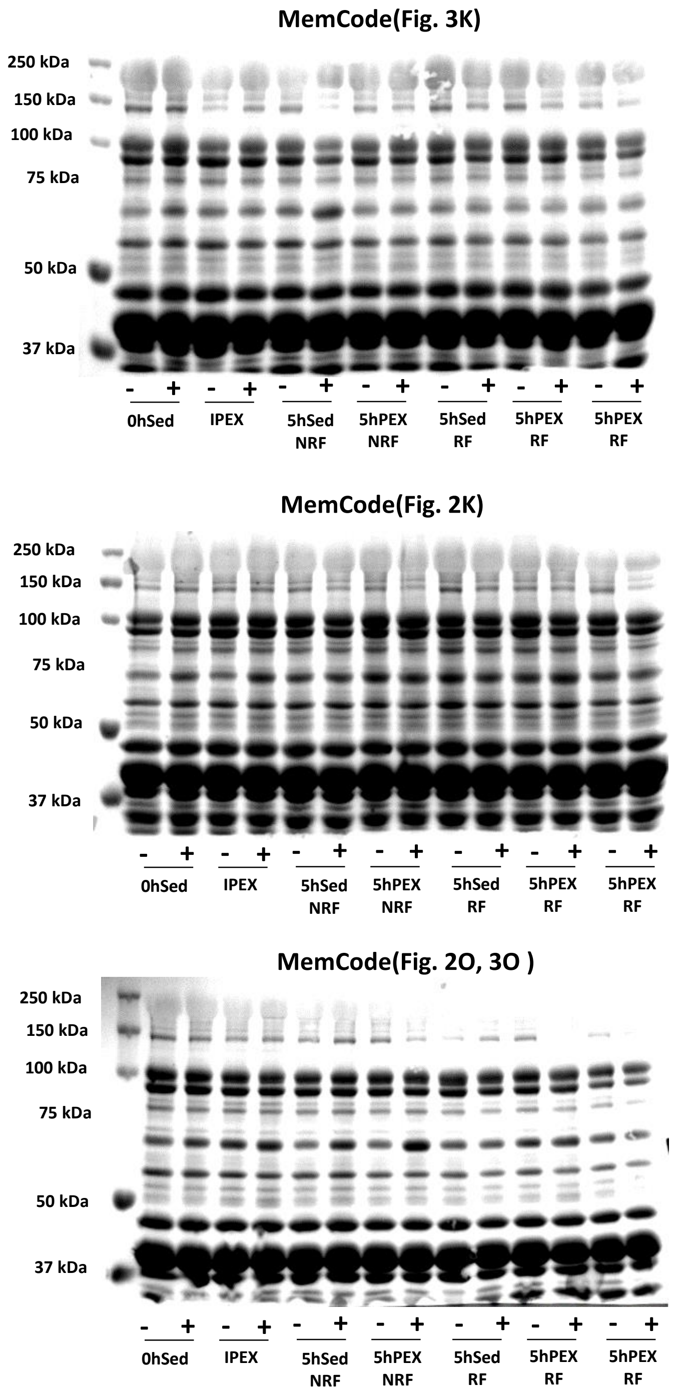

+:shRNA-GS1 injected muscle
-:shRNA-Scr injected muscle
All imiages were captured using the Chemiluminescence channel by FluorChemE by Proteinsimple

Figure 4 raw images

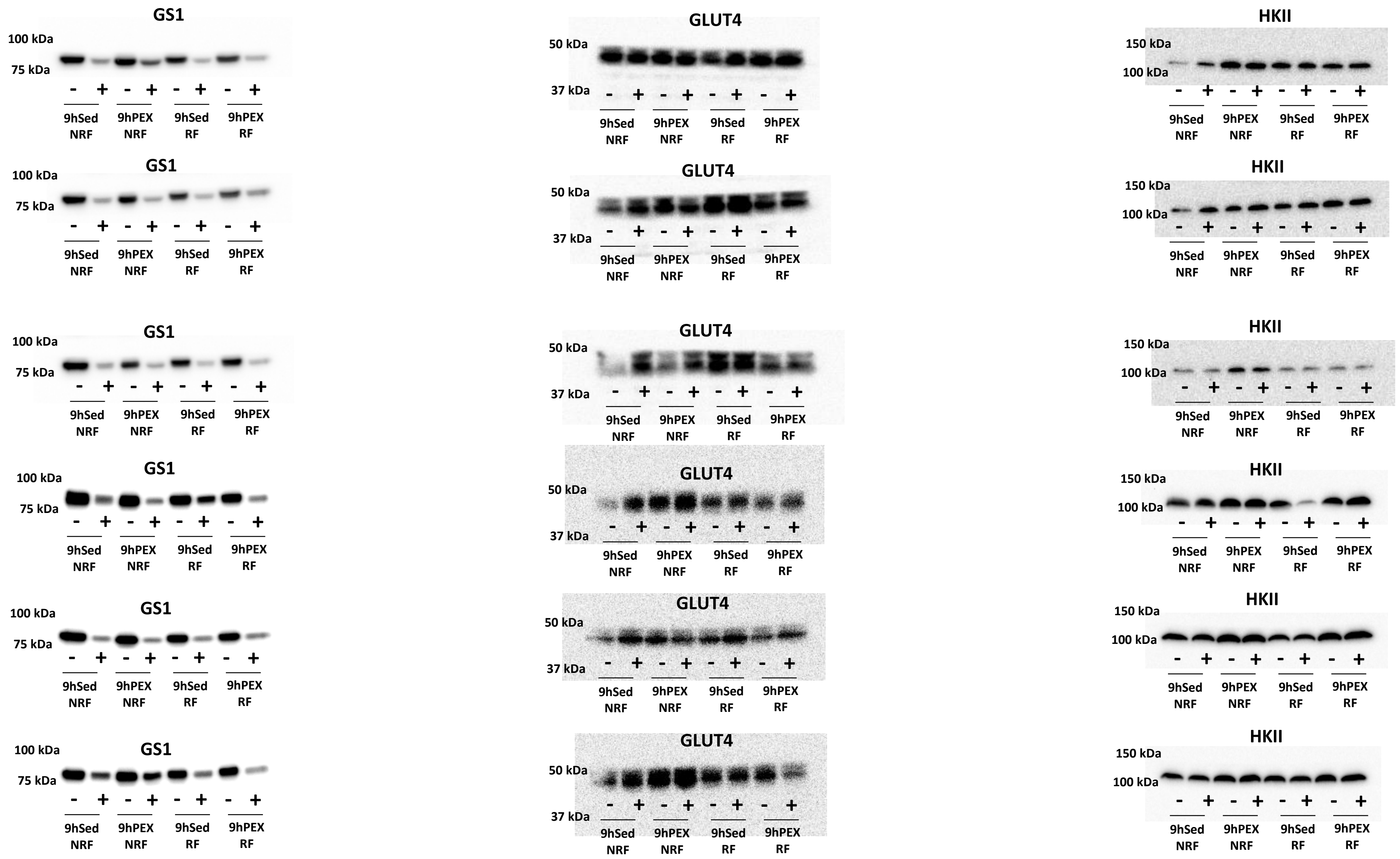

+:shRNA-GS1 injected muscle

-:shRNA-Scr injected muscle

All imiages were captured using the Chemiluminescence channel by FluorChemE by Proteinsimple

Figure 4 raw images

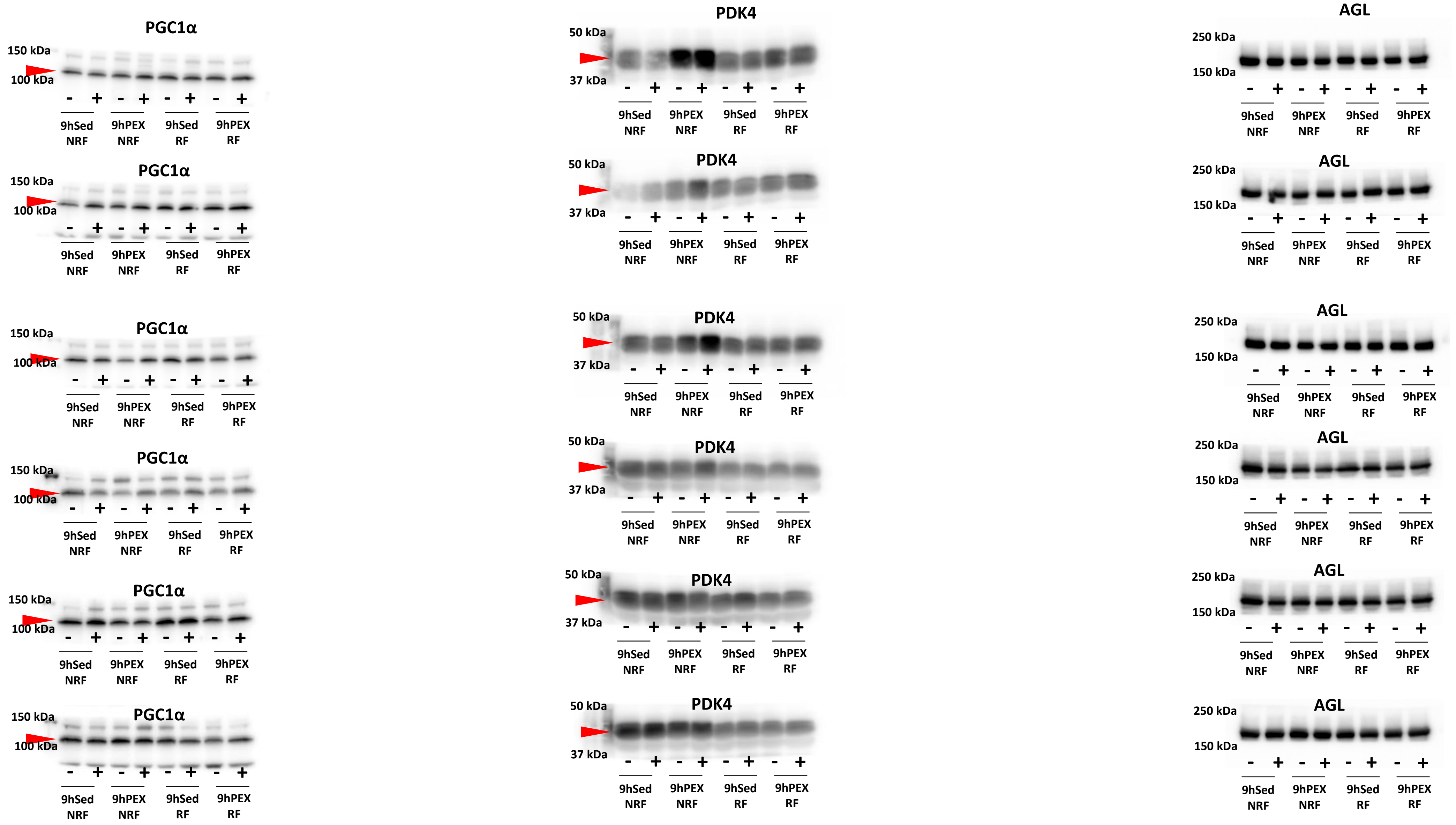

+:shRNA-GS1 injected muscle

-:shRNA-Scr injected muscle

All imiages were captured using the Chemiluminescence channel by FluorChemE by Proteinsimple

Denotes quantified band at expected molecular weight

Figure 4 raw images

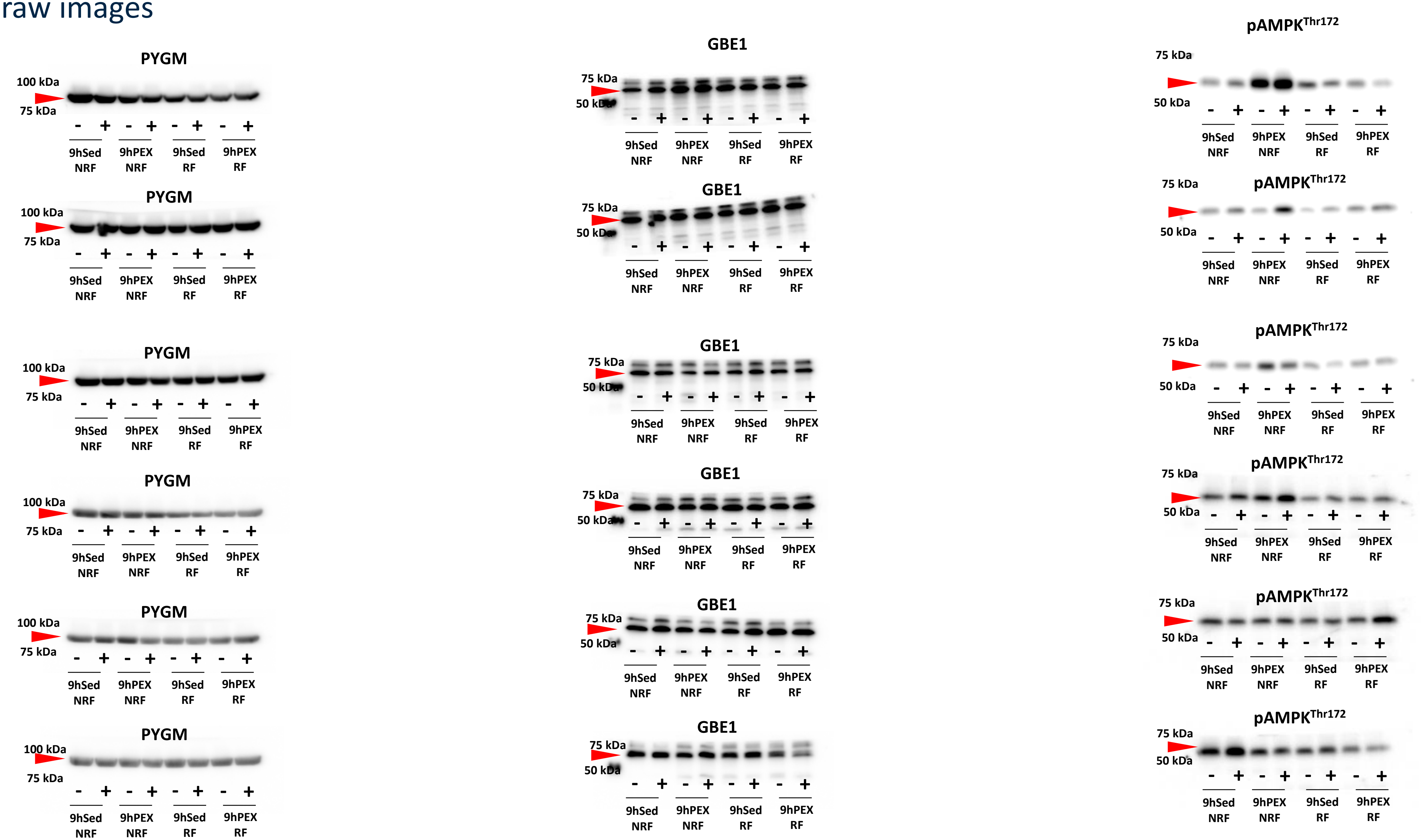

+:shRNA-GS1 injected muscle

-:shRNA-Scr injected muscle

All imiages were captured using the Chemiluminescence channel by FluorChemE by Proteinsimple

Denotes quantified band at expected molecular weight

Figure 4 raw images

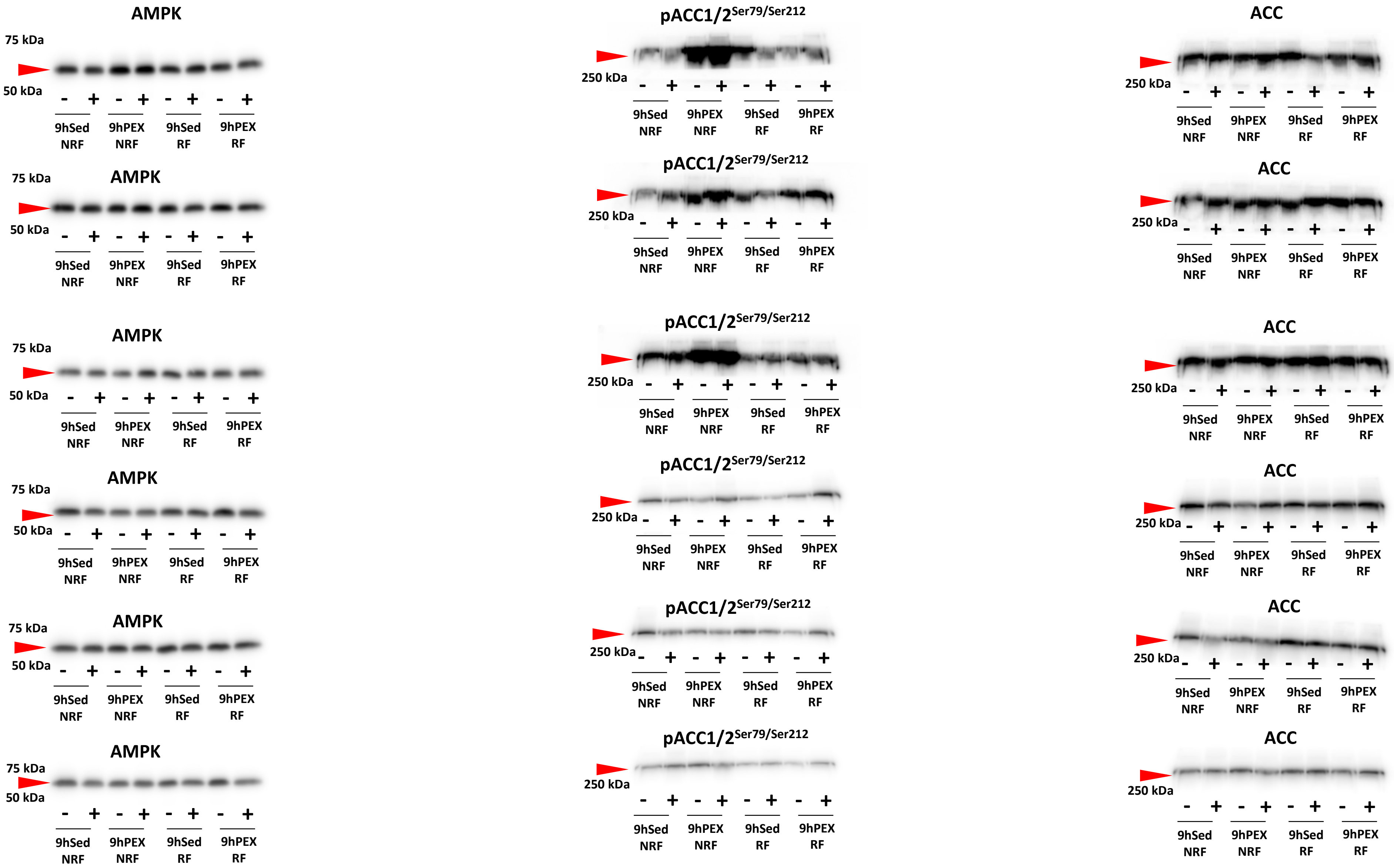

+:shRNA-GS1 injected muscle

-:shRNA-Scr injected muscle

All imiages were captured using the Chemiluminescence channel by FluorChemE by Proteinsimple

Denotes quantified band at expected molecular weight

Figure 4 raw images

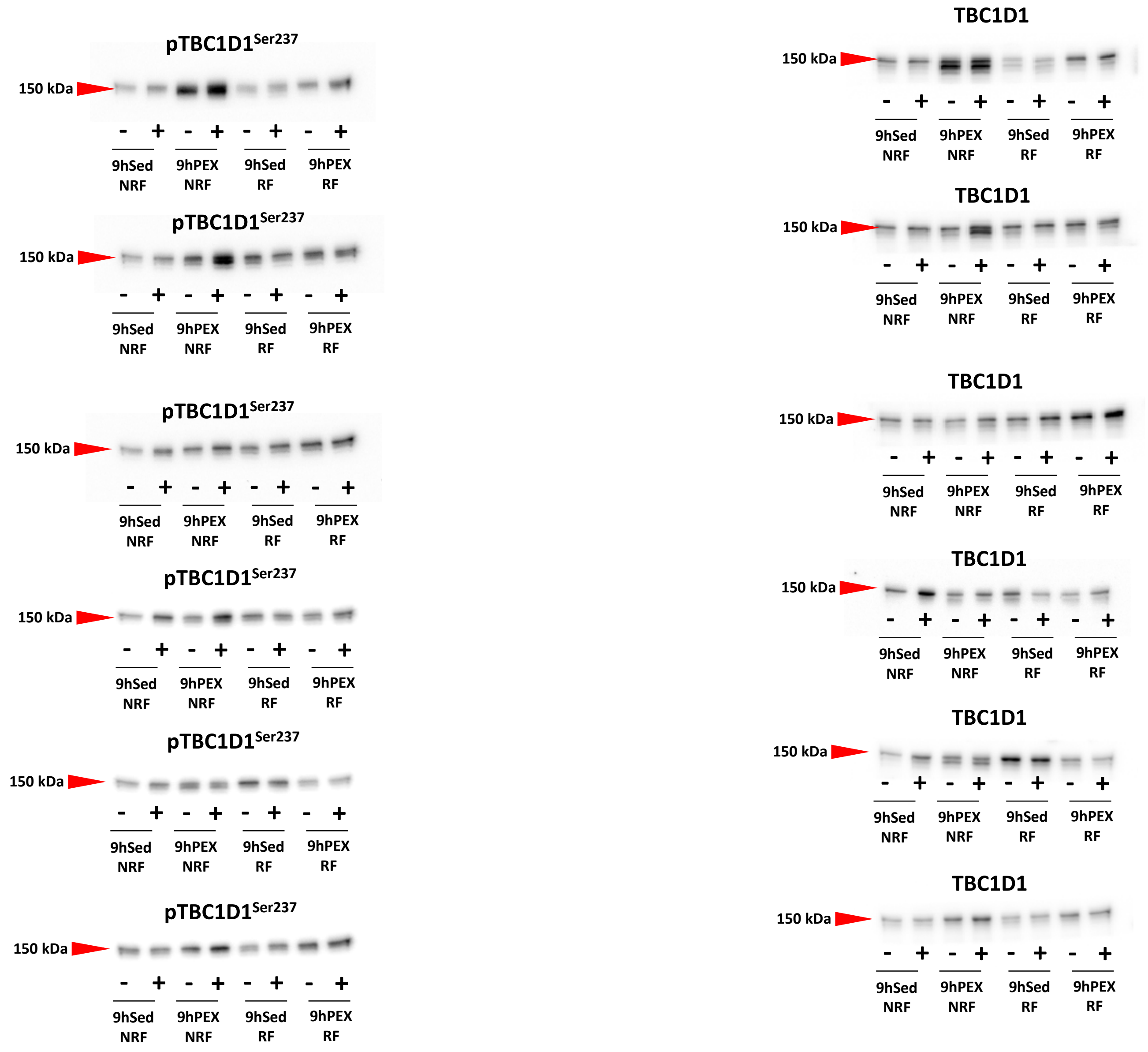

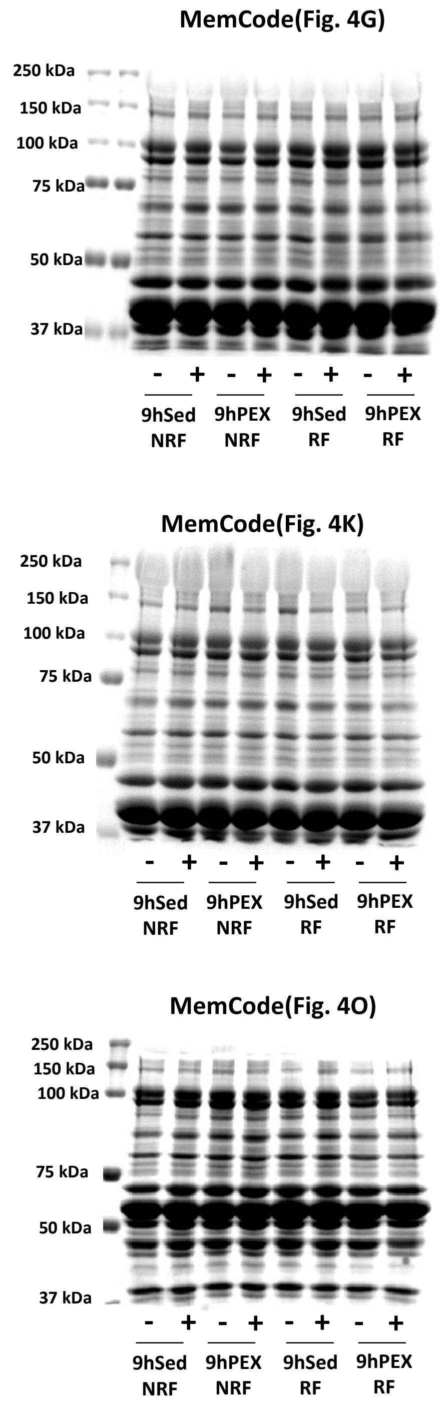

+:shRNA-GS1 injected muscle

-:shRNA-Scr injected muscle

All imiages were captured using the Chemiluminescence channel by FluorChemE by Proteinsimple

Denotes quantified band at expected molecular weight
